# A Well-Defined Readily Releasable Pool with Fixed Capacity for Storing Vesicles at Calyx of Held

**DOI:** 10.1371/journal.pcbi.1004855

**Published:** 2016-04-01

**Authors:** Kashif Mahfooz, Mahendra Singh, Robert Renden, John F. Wesseling

**Affiliations:** 1 Department Neurociencias (CIMA), Universidad de Navarra, Pamplona, Spain; 2 Department of Physiology & Cell Biology, University of Nevada School of Medicine, Reno, Nevada, United States of America; Indiana University, UNITED STATES

## Abstract

The readily releasable pool (RRP) of vesicles is a core concept in studies of presynaptic function. However, operating principles lack consensus definition and the utility for quantitative analysis has been questioned. Here we confirm that RRPs at calyces of Held from 14 to 21 day old mice have a fixed capacity for storing vesicles that is not modulated by Ca^2+^. Discrepancies with previous studies are explained by a dynamic flow-through pool, established during heavy use, containing vesicles that are released with low probability despite being immediately releasable. Quantitative analysis ruled out *a posteriori* explanations for the vesicles with low release probability, such as Ca^2+^-channel inactivation, and established unexpected boundary conditions for remaining alternatives. Vesicles in the flow-through pool could be incompletely primed, in which case the full sequence of priming steps downstream of recruitment to the RRP would have an average unitary rate of at least 9/*s* during heavy use. Alternatively, vesicles with low and high release probability could be recruited to distinct types of release sites; in this case the timing of recruitment would be similar at the two types, and the downstream transition from recruited to fully primed would be much faster. In either case, further analysis showed that activity accelerates the upstream step where vesicles are initially recruited to the RRP. Overall, our results show that the RRP can be well defined in the mathematical sense, and support the concept that the defining mechanism is a stable group of autonomous release sites.

## Author Summary

Short-term plasticity has a dramatic impact on the connection strength of almost every type of synapse during normal use. Some synapses enhance, some depress, and many enhance or depress depending on the recent history of use. A better understanding is needed for modeling information processing in biological circuits and for studying the molecular biology of neurotransmission. Here we show that first principles at the calyx of Held, such as whether or not a readily-releasable pool of vesicles in the presynaptic terminal has a fixed capacity for storing vesicles, are unexpectedly similar to synapse types that are used at much lower frequencies. Our study establishes new methods for studying the function of presynaptic molecules, and the results suggest that a tractable general model of short-term plasticity can capture the full computational power of dynamic synaptic modulation across a large range of synapse types and situations.

## Introduction

The readily releasable pool (RRP) of vesicles is a reference concept for studies of presynaptic function. The concept was originally proposed to explain quantitative relationships between the frequency of presynaptic action potentials and short-term depression at neuromuscular junctions [1], but has since been used as a framework for a wide variety of central synapses. The current idea is that only a few *per cent* of vesicles in typical presynaptic terminals are ready to release at any given time and that at least some readily releasable vesicles are morphologically docked to the active zone and primed for release [2].

Such an organization suggests that presynaptic function might be determined by the aggregate behavior of a fixed population of stable, autonomous release sites [3–6]. The concept of a fixed population of release sites was never proven, but fits well with a wide assortment of results from excitatory hippocampal synapses [7–11].

However, the molecular biology of synaptic vesicle trafficking seems to be complicated, and at least one attempt at a comprehensive model of short-term plasticity has questioned the utility of the RRP as a useful premise [12]. More concretely, the idea that the RRP has a fixed capacity for storing vesicles is fundamental to the concept as originally envisioned [1, 4]. And yet, estimates of RRP size at calyx of Held synapses in the medial nucleus of the trapezoid body (MNTB) in the brain stem vary at least 5-fold between studies, and experimental details that should be irrelevant, such as the level of extracellular Ca^2+^, seem to play a key role [13–16]. On the other hand, the RRP seems to have a well-defined size at hippocampal synapses; the Ca^2+^-dependence of transmitter release at hippocampal synapses is instead wholly because Ca^2+^ controls the efficiency of the coupling between action potentials and transmitter release [7, 9, 10].

The reasons for differences between calyces of Held and hippocampal synapses are not clear. The extracellular Ca^2+^ level seems to be most relevant when RRP size is estimated from the post synaptic responses evoked by trains of presynaptic action potentials, but less relevant—or not relevant—when neurotransmitter release is driven by briefly voltage clamping the presynaptic terminal at depolarized potentials [17]. Voltage clamp depolarization depletes the RRP in 10’s of *ms*, and potentially involves washing out endogenous solutes that might be important for second messenger signaling. In contrast, trains of action potentials require 100’s of *ms*, but can be evoked without altering the intracellular milieu. Thus, sustained activity might trigger an expansion of RRP capacity at the calyx of Held *via* second messenger mechanisms not present at hippocampal synapses [11, 18].

On the other hand, currently available information about calyces of Held was extracted using experimental techniques that may not be directly comparable to the techniques used at hippocampal synapses, and it is possible that operating principles are more similar at the level of basic mechanisms than presently thought. For example, an alternative explanation for the apparent Ca^2+^ dependence of RRP size might be that action potential trains thought to be maximal were not sufficient to completely exhaust the RRP at the lower Ca^2+^ levels [19].

Here we use a variety of fiber stimulation protocols at calyces of Held from 14–21 days postnatal mice to show that, indeed, the RRP capacity for storing vesicles is not influenced by extracellular Ca^2+^, even during trains of action potentials lasting 100’s of *ms*. Discrepancies with previous studies are explained by the presence during 100*Hz* stimulation of a standing flow-through pool of vesicles that are reluctant to release because of low release probability, but that are nevertheless immediately releasable. A quantitative analysis of the results demonstrated that the RRP concept can be well-defined, in the mathematical sense, in a way that is largely compatible with the original ideas in [1, 3].

Vesicles with low release probability were not envisioned in the original conceptualization of the RRP, but could be explained by several competing hypotheses that have been proposed more recently. Either vesicle priming could be sequential, in which case vesicles with low release probability would be in an immature state of priming [20–22]. Or, vesicles with low and high release probability could be recruited in parallel to separate types of release sites [23]. Our results do not determine which explanation is correct, but do yield unexpectedly fast limits on sequential priming models, and show that parallel models are mathematically more parsimonious.

## Results

Our initial goal was to compare the operating principles of vesicle trafficking at calyces of Held to previous results from excitatory hippocampal synapses. To accomplish this, we recorded EPSC responses in MNTB neurons while evoking trains of action potentials in the afferent axon. This is an extensively studied monosynaptic connection where the calyx of Held is the presynaptic terminal [24]. The calyces were intact in that the intracellular milieu was not altered by patch-clamping.

The experimental paradigm required repetitive afferent stimulation at frequencies that were high enough to either completely empty the RRP, or to drive it to a steady state level of fullness where recruitment of new vesicles was balanced by release of vesicles that were ready [9]. Although 20*Hz* trains were sufficient to exhaust the RRP at hippocampal synapses [9, 11], we show below that even 100*Hz* was not sufficient at the calyx of Held, but that 300*Hz* was sufficient.

We did observe intermittent transmission failures during 300*Hz* stimulation in a substantial minority of preparations (perhaps one-third of preparations had failures during trains of 300*ms*). The failures likely arose from failures of action potential initiation or conduction in the afferent axon because they often occurred regularly after every second or third pulse of stimulation, and because the response sizes during successful transmission were large compared to the size of miniature spontaneous EPSCs. Preparations exhibiting failures were discarded. Action potential failures were not a concern for the remaining experiments because individual MNTB neurons receive glutamatergic input from a single axon/calyx.

**Fig 1:**
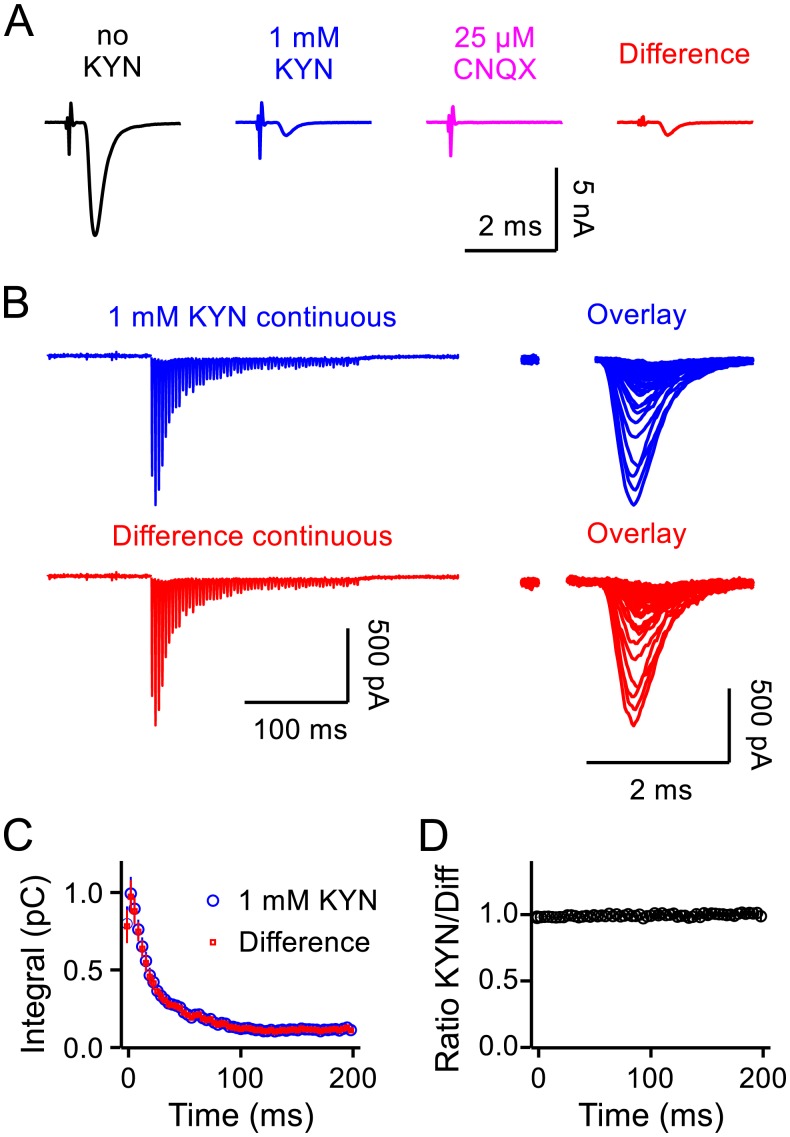
Stimulus artifact elimination. **A.** Sequence of examples of isolated EPSCs without KYN, in 1*mM* KYN, in 25*μM* CNQX, and the difference trace obtained by digitally subtracting the recording in CNQX from the recording in 1*mM* KYN. **B.** EPSCs during 300*Hz* stimulation; artifacts were removed by blanking a window of 1*ms* for traces in 1*mM* KYN and 0.5*ms* for the difference traces. **C.** Integral of sequential 3.33*ms* segments *vs* time of stimulation for traces in 1*mM* KYN and of the difference after subtracting matching traces in CNQX (*n* = 5 preparations). **D.** Ratio of corresponding integrals for traces in 1*mM* KYN and the difference traces *vs* time of stimulation.

The experimental paradigm additionally required a linear relationship between neurotransmitter release and postsynaptic response. To accomplish this, experiments were conducted in 1 or 2*mM* kynurenic acid (KYN), which blocks the response 87 ± 0.8%(*n* = 26; Fig 1A) and 94 ± 0.3%(*n* = 22) respectively, and eliminates postsynaptic mechanisms of short-term plasticity such as receptor desensitization and saturation [25]. NMDA-type glutamate receptors were blocked completely with APV throughout.

### Control for distortions caused by stimulus artifacts

A primary aim was to monitor the changes in the rate of release that occur during train stimulation, including changes in so-called asynchronous release which is not tightly synchronized to individual action potentials [19]. To achieve this, recordings were acquired and digitized without gaps, and baselines were calculated from the 100*ms* interval preceding stimulation trains (Fig 1B, upper left trace) instead of the more usual method of calculating the baseline separately for individual responses.

Stimulus artifacts were removed over windows lasting 1*ms* (Fig 1B, upper right), which could have been problematic if the artifacts included long-lasting tails that extended outside of the window that was removed. To control for this, we calculated difference traces by subtracting traces recorded in 25*μM* CNQX from the traces in 1*mM* KYN. The procedure yields pure AMPA-type glutamate receptor responses without stimulus artifacts because CNQX is an AMPA receptor antagonist (Fig 1A). In practice, a small residual component of the stimulus artifact remained in the difference traces (Fig 1A, red trace), but could be eliminated by removing a narrower window of 0.5*ms* or less (Fig 1B, red trace), and even when not eliminated did not contribute to measurements of the current integral because the positive and negative components canceled each other.

Comparisons between responses from the difference traces and traces in 1*mM* KYN showed that any distortions caused by stimulus artifacts were not significant if present at all (Fig 1C and 1D). This control experiment additionally ruled out non-linear contributions of glutamate uptake currents and ephaptic transmission; glutamate uptake currents were already shown to be absent from principal neurons of the MNTB at similar developmental stages in rats [26].

EPSCs during 300*Hz* stimulation depressed to a low steady state size within 100*ms* (30 action potentials; Fig 1C). The timing of depression was much slower than RRP depletion driven more directly by photolytic Ca^2+^ uncaging within patch-clamped calyces, suggesting that transmitter release was not rate-limited by molecular constraints on the release machinery but by the frequency of action potentials [24].

### No Ca^2+^ modulation of RRP size

RRP size at hippocampal synapses does not seem to be influenced by extracellular Ca^2+^ levels, but the situation might be different for the calyx of Held. More specifically: the amount of neurotransmitter released by individual action potentials is well-known to increase when extracellular Ca^2+^ is increased at every synapse type, but—at least at hippocampal synapses—the total amount released by procedures that exhaust the RRP is constant. In contrast, some procedures thought to exhaust the RRP or an immediately releasable subdivision at the calyx of Held released more neurotransmitter when extracellular Ca^2+^ was higher, suggesting that elevating Ca^2+^ increases the capacity for storing vesicles [13, 15]. Such results might indicate a qualitative difference between calyces and hippocampal synapses. However, an alternative explanation with some already published support would be that the 100*Hz* stimulation used to elicit release in the previous studies was not sufficient to exhaust the RRP completely at standard Ca^2+^ levels [17, 19].

**Fig 2:**
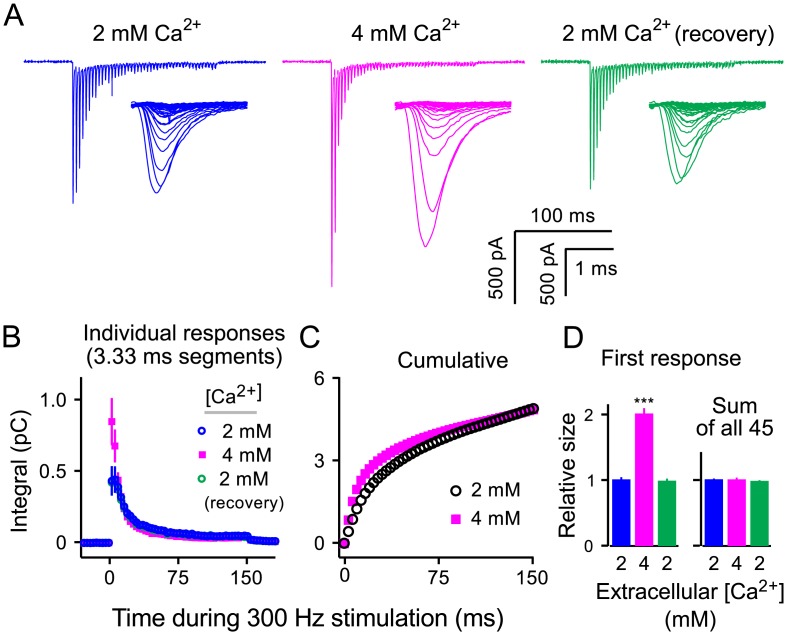
Larger initial responses are balanced by faster induction of depression in high Ca^2+^ when stimulation is 300*Hz*. **A.** Example traces recorded in 2*mM* Ca^2+^, 4*mM* Ca^2+^, and again in 2*mM* Ca^2+^; each trace is the average of 3 consecutive trials; KYN was 2*mM*; stimulus artifacts are blanked. The mechanism causing the rightward shifts in the time courses as individual EPSCs become smaller have been investigated elsewhere [27]. **B.** Integrated 3.33*ms* segments corresponding to the interval between action potentials *vs* time (*n* = 5). **C.** Cumulative plot of integrated responses showing that the total amount of release was the same; the symbols corresponding to 2*mM* Ca^2+^ (black circles) are the average of the blue and green symbols from **Panel (B)**. **D.** Summary data showing relative sizes of 1^st^ responses (left), and integrals of all 45 responses (right; *p* < 0.001, Kolmogorov-Smirnov).

To determine if estimates of RRP capacity continue to depend on extracellular Ca^2+^ when stimulation was 3-fold faster, we compared the sum of postsynaptic responses during 300*Hz* stimulation (45 presynaptic action potentials in 150*ms*) in 2*mM*, 4*mM*, and again in 2*mM* Ca^2+^ (Fig 2A); KYN was 2*mM* throughout. Trials were conducted in sets of 3 identical repetitions, with interleaved rest intervals of 1*min*, and the digitized traces for each set were averaged together before further analysis. Stimulus artifacts were removed as above and data were only accepted for further analysis if the sum of EPSCs recovered to within 5%; 2 of 7 preparations were discarded because reversal was not achieved.

For analysis, a global baseline was subtracted as for Fig 1C and 1D and traces were divided into 45 sequential 3.33*ms* segments corresponding to the interval between pulses of stimulation. Responses were then quantified by calculating the integral of each segment (Fig 2B).

The response to the first pulse of stimulation was 2.0 ± 0.1-fold larger (*n* = 5) in 4*mM* Ca^2+^ and subsequent depression occurred more rapidly so that the responses to pulses 15–30 were slightly smaller (Fig 2B, magenta). However, the cumulative response was not different in 4*mM* Ca^2+^compared to in 2mM (*i.e.*, nominally 1.02 ± 0.02-fold larger; Fig 2C and 2D). This result confirms that 300*Hz* stimulation is sufficient to exhaust the entire RRP when extracellular Ca^2+^ is 2*mM*, and suggests that the capacity of the RRP for storing vesicles is constant; we emphasize that the RRP measured here includes both slow- and fast-releasing subdivisions.

### Quantal content of RRP after long rest intervals

We estimated that the RRP at the start of stimulation contained a mean of 2553 ± 343(*n* = 18) synaptic vesicles; the coefficient of variation across preparations was 55%. The estimate was calculated by dividing the cumulative response during 300*Hz* stimulation by the average charge transfer of spontaneous miniature EPSCs (mEPSCs; 31.4 ± 2.1*fC*). The cumulative response was first corrected for the recruitment of new vesicles to the RRP during ongoing stimulation using Eqs 1 and 2 introduced below as part of a more detailed analysis; estimates generated using the back extrapolation method developed in [13] produced slightly lower values, but the more detailed analysis explains why using Eqs 1 and 2 is likely more accurate. The mEPSCs were measured over 10–30*s* of continuous recording before adding KYN and starting the experiment, and were therefore scaled by 0.13 for experiments conducted in 1*mM* KYN or 0.06 for experiments in 2*mM* KYN; preparations where the smallest mEPSCs could not be distinguished easily from noise were excluded. The mean quantal content and variation were similar to previous estimates from patch-clamped calyces where release was elicited ∼10-fold more quickly by step depolarizations that allowed massive Ca^2+^ influx *via* voltage gated ion channels [17].

### Persistence of readily releasable vesicles during 100 Hz stimulation

The results of the quantal content analysis thus fit well with the idea that the RRP measured with trains of action potentials at 300*Hz* is the same quantity released by presynaptic step depolarizations. The studies using step depolarizations showed that once initiated, the rate of release does not decay away with a single exponential time course as would be expected if all readily-releasable vesicles undergo exocytosis with the same probability of release (hereafter denoted by *p*_*v*_ for probability of release per available vesicle within the RRP). Instead, the time course has multiple phases, which motivated the current concept that the RRP is made up of distinct slow-releasing and fast-releasing subdivisions; slow- and fast-releasing subdivisions have previously been termed SRP for Slow Releasing Pool and FRP for Fast Releasing Pool [28]. We therefore reasoned that the greater amount of release in elevated *vs* standard Ca^2+^ seen previously when action potential trains were 100*Hz*, and confirmed below, would be consistent with the results in Fig 2 if 100*Hz* was not intense enough to completely empty a slow-releasing subdivision of the RRP at the standard Ca^2+^ level. To explore this possibility and related alternatives, we performed frequency jump experiments where the frequency of stimulation was abruptly increased to 300*Hz* after inducing a steady state level of depression at 100*Hz*. Frequency jump experiments have been conducted previously at the calyx of Held, but at lower frequencies for a different purpose [29]; however, see [9, 11] for frequency jump experiments conducted for the same purpose, but at hippocampal synapses.

**Fig 3:**
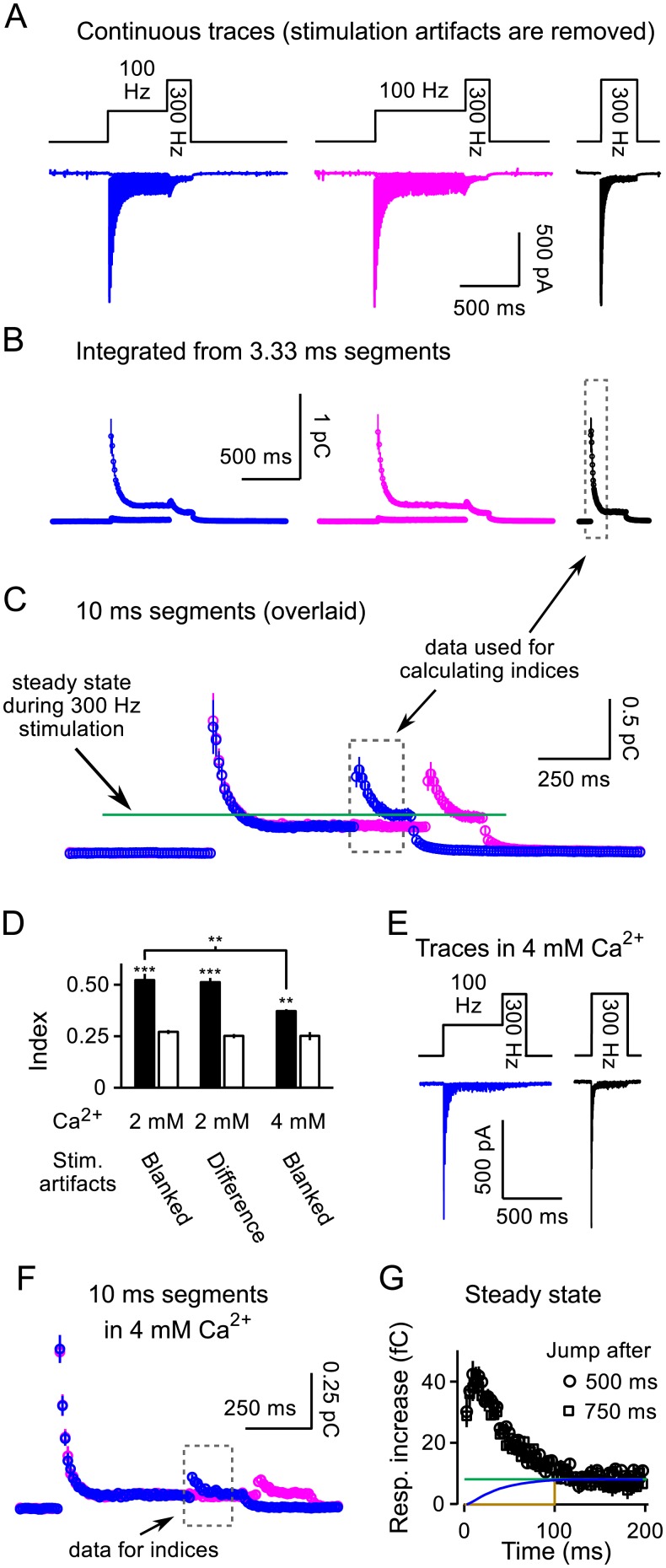
Frequency jump experiments. **A.** Average traces from *n* = 16 preparations (3 trials per preparation) where the stimulation frequency was abruptly increased to 300*Hz* after either 500*ms* (blue) or 750*ms* (magenta) of 100*Hz* stimulation. Black trace is 200*ms* of 300*Hz* stimulation from the same preparations. Stimulus artifacts were blanked. Ca^2+^ was 2*mM* throughout; KYN was 1*mM*. 4 preparations included in the full analysis in the Results were excluded from these display traces because stimulation during 300*Hz* stimulation was 150*ms* instead of 200*ms*. **B.** Overlaid integrals from 3.33*ms* segments. The double lines during 100*Hz* stimulation are because 23 of segments contained only asynchronous responses whereas every 3^rd^ contained a synchronous response, which was larger. **C.** Overlaid integrals of 10*ms* segments revealing a robust increase in the release rate caused by increasing stimulation to 300*Hz*.**D.** Indices of increase in response caused by frequency jumps (see Results). Solid bars are for trials where stimulation frequency was increased to 300*Hz*, open bars are matched baseline values where stimulation was instead maintained at 100*Hz*. **E.** Average of traces from *n* = 3 preparations from frequency jump experiments where extracellular Ca^2+^ was 4*mM*. KYN was 2*mM*, explaining why the initial response sizes were not larger than in **Panel (A)**. **F.** Integrals of segments for experiments in 4*mM* Ca^2+^analogous to **Panel (C)**.**G.** Response increase after frequency jumps initiated after 500 or 750*ms* of 100*Hz* stimulation in 2*mM* Ca^2+^ (*n* = 7; segments were 3.33*ms*). The baseline response size during matched 100*Hz* stimulation was calculated by averaging three consecutive 3.33*ms* segments to smooth out the variation between sequential segments seen in **Panel (B)**, and was subtracted beforehand. Colored lines are estimates of vesicle recruitment to sites newly vacated by the frequency jump: blue assumes Eq 1 with ${\hat{\alpha }}_{fixed}=4.33/s$; green is the overestimate where bulk refilling is maximal from the start; brown is the underestimate where recruitment does not begin until all release sites have been vacated (see Results).

Sequential data processing is shown in Fig 3A–3C for interleaved trials where frequency jumps were initiated after both 500*ms* (blue) and 750*ms* (magenta) of 100*Hz* stimulation; we additionally interleaved trials where stimulation was 300*Hz* for 200*ms* for later comparisons (Fig 3A and 3B, black). Values plotted in Fig 3B were obtained by integrating over sequential segments of 3.33*ms* duration. Only every third segment contained synchronous responses during 100*Hz* stimulation because the inter-stimulus interval was 10*ms*; the smaller values making up the lower of the double horizontal lines are measures of the asynchronous component of responses occurring more than 3.33*ms* after the individual pulses of stimulation.

Plotted this way, individual responses can be seen to depress to a first plateau during 100*Hz* stimulation, and then to a second plateau that is lower during subsequent 300*Hz* stimulation. The result indicates that the quantity of neurotransmitter release elicited by individual action potentials was less at 300*Hz*, in-line with the expectation that rate-limiting steps in recruitment of new vesicles to the RRP played a role after 500*ms* of 100*Hz* stimulation [13].

The values in Fig 3C were obtained by integrating over segments of 10*ms* instead of 3.33*ms*, which provides a more direct comparison of release as a function of time for stimulation at 100 *vs* 300*Hz*. Direct comparisons are valid because stimulus artifacts were eliminated by removing 1*ms* windows every 3.33*ms*, even for the baseline and segments during 100*Hz* stimulation. The values of the segment integrals were larger after switching to 300*Hz* stimulation than during 100*Hz* stimulation—even though the release *per* individual action potential was less—because each segment contained responses to 3 action potentials.

To quantify the increase without making assumptions about mechanism, we calculated an index by dividing the sum of values from the first 150*ms* after increasing the stimulation frequency (blue points in dashed box in Fig 3C) by the sum of values from the first 150*ms* of the trials where stimulation was 300*Hz* throughout (box in Fig 3B; leftmost solid bar in Fig 3D; *n* = 20). A baseline value for the index was calculated by dividing the sum of the matching values from trials where the stimulation was maintained at 100*Hz* (magenta points in dashed box in Fig 3C) by the sum of points in the box in Fig 3B. The baseline value (leftmost open bar in Fig 3D) was significantly less than for frequency jumps, confirming that increasing the stimulation frequency to 300*Hz* increased the rate of release.

Stimulus artifacts did not play a role because an identical analysis of difference traces calculated as in Fig 1 produced a similar result (third and fourth bars in Fig 3D). Difference traces were only available for a subset of preparations; for these, experimental trials were followed with matched trials in the presence of either 4*mM* KYN (*n* = 3) or 25*μM* CNQX (*n* = 4).

We additionally conducted analogous frequency jump experiments in 4*mM* Ca^2+^ (Fig 3E and 3F). The idea was that increasing extracellular Ca^2+^ would increase the fraction of the RRP released by individual action potentials, which would lead to more RRP depletion. As predicted, the increase in release elicited by frequency jumps was less (compare Fig 3F to Fig 3C); the index of increase was midway between the baseline value and the value in 2*mM* Ca^2+^(Fig 3D, compare bars 1 and 5). The indices are directly comparable because of the result, above, that the time-integrated response during the first 150*ms* of 300*Hz* trials was the same in 2*mM* and 4*mM* Ca^2+^ (see Fig 2C and 2D). This result confirms that the increase in release elicited by frequency jumps is caused by release of transmitter from a readily-releasable supply that was not released during 100*Hz* stimulation.

Most of the increase in the rate of release seen at both 2 and 4*mM* Ca^2+^ was transient, confirming that 100*Hz* stimulation leaves a residual supply of readily releasable vesicles that can be induced to undergo exocytosis by increasing the frequency to 300*Hz*. Further analysis using Eqs 1 and 2 introduced below indicated that 100*Hz* stimulation depleted the RRP: 87% ± 3% when Ca^2+^ was 4*mM*; 79% ± 3% when Ca^2+^ was 2*mM*; and 61% ± 8%(*n* = 4) when Ca^2+^ was 1.2*mM*, which is at or below the level *in vivo*[30] and the lower level used in [15] (see S1 Fig).

The rate of release during the frequency jump experiments did reach a new steady state after 120*ms* of 300*Hz* stimulation that was elevated compared to the steady state during 100*Hz* stimulation. The elevated steady state suggests that bulk recruitment of new vesicles was faster during 300*Hz* stimulation (Fig 3C, green line). The elevation is in line with multiple mechanisms, including a likely increase in the number of vacancies within the RRP and possible activity-dependent acceleration of the mechanism underlying vesicle recruitment (see **Lemma 7: Second order corrections**, which is introduced below as part of the more detailed analysis).

### Flow-through pool concept

The amount of increase was similar when the frequency jump was initiated 250*ms* later, after 750*ms* of 100*H*z stimulation (96 ± 3% when Ca^2+^ was 2*mM*; Fig 3G). For this comparison, increases were calculated after subtracting the responses during matched trials where the stimulation was maintained at 100*Hz* for the full 950*ms*, which was necessary for a high precision analysis because of slowly-developing fatigue in recruitment of new vesicles, documented below. 100*Hz* trials matching frequency jumps after both 500 and 750*ms* were available for *n* = 7 preparations, all in 2*mM* Ca^2+^; for these, trials of 100*Hz* stimulation lasting 950*ms* were interleaved with the two types of frequency jumps and 300*Hz* trials.

Thus, the presence of the unreleased supply was not simply because 50 action potentials at 100*Hz* were too few to exhaust the slow-releasing subdivision of the RRP. Instead, the RRP was maintained at a steady-state level of fullness. This could either be because readily releasable vesicles constituted a flow-through pool where recruitment of new vesicles is fast enough to balance the quantity undergoing exocytosis. Or, the steady state supply could be completely immobile when stimulation is 100*Hz*, and only accessed for release when the frequency is increased to 300*Hz*. However, correlations presented below between paired pulse facilitation/depression and the size of the steady-state supply seem to argue against the hypothesis that vesicles remaining within the RRP during steady state 100*Hz* stimulation are immobile, and therefore support the concept of a flow-through pool.

### Steady state vesicles have low *p*_*v*_

**Fig 4:**
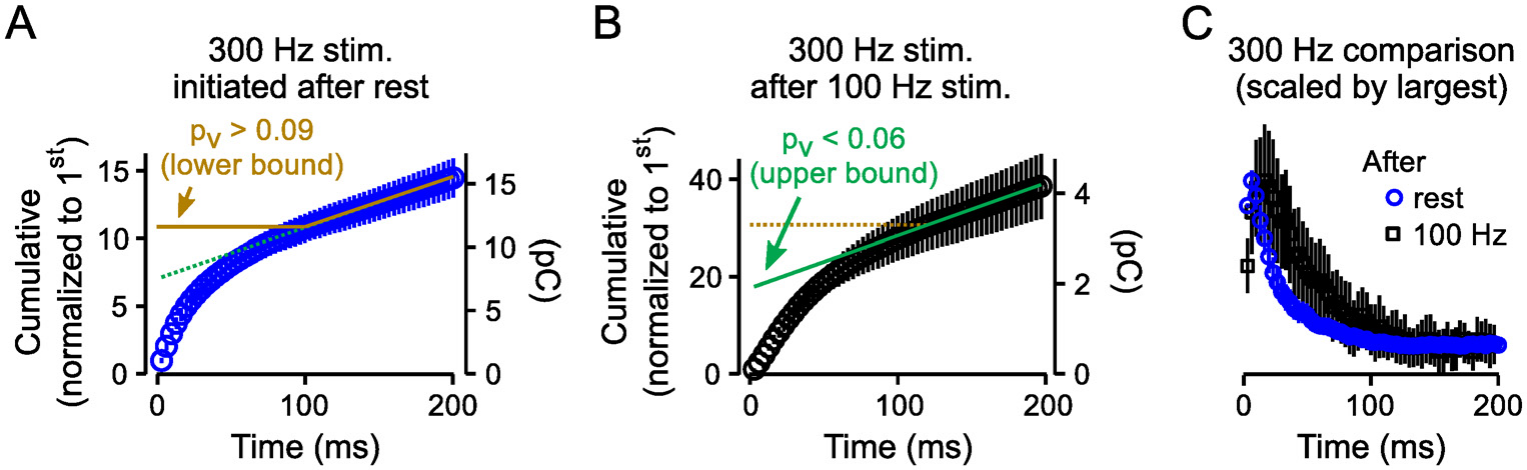
Higher *p*_*v*_ when 300*Hz* stimulation was initiated after rest *vs* after 100*Hz* stimulation. The main point is that the mean value for *p*_*v*_ for vesicles within the flow-through pool during 100*Hz* stimulation is less than the mean for vesicles in the RRP at the start of stimulation (*n* = 20 preparations; data points were quantified from segments of 3.33*ms* throughout). **A**. Cumulative plot of time-integrated segments (3.33*ms*) when 300*Hz* stimulation was initiated after rest. Brown and green lines match steady state responses attributed to release of newly recruited vesicles. The brown line is back-extrapolated assuming new vesicle recruitment began only after 100*ms* of stimulation, making the intercept at *Time* = 0 an overestimate of RRP capacity. The y-axis on the left was calibrated so that the first response was 1.0, making the reciprocal of the intercept a lower bound estimate for the mean value of *p*_*v*_ for all vesicles within the RRP when fully replenished. The green line is back-extrapolated assuming that the bulk recruitment rate was constant from *Time* = 0; in this case, the reciprocal of the left y-axis intercept is an upper bound for *p*_*v*_. The y-axis on the right is calibrated in absolute units (*pC*) to facilitate comparisons with **Panel (B)**. **B.** Cumulative plot of segments when 300*Hz* stimulation was initiated following 500*ms* of 100*Hz* stimulation. The matching response rate from interleaved trials when stimulation was 100*Hz* was subtracted beforehand. The y-axis on the left was calibrated so that the first response during 300*Hz* stimulation was 1.0, making the reciprocal of the *Time* = 0 intercept (green line) an upper bound for *p*_*v*_ of vesicles within the RRP immediately before increasing the stimulation frequency to 300*Hz*; the first response during 300*Hz* stimulation is the last response during 100*Hz* stimulation. The calibration of the left y-axis is not directly comparable to the left y-axis in **Panel (A)**, but the right y-axes are directly comparable. **C.** Scaled response sizes during 300*Hz* stimulation after rest and after frequency jumps; steady state values were subtracted before scaling.

We found that the mean value for *p*_*v*_ for vesicles in the steady state supply was lower than the mean value when the RRP was full. To demonstrate the difference in a way that does not depend on assumptions about ongoing vesicle recruitment, we estimated a lower bound for the mean *p*_*v*_ for all of the vesicles in the RRP at the start of stimulation (Fig 4A, reciprocal of y-axis intercept of brown line) that was higher than an upper bound for the mean value for vesicles in the steady-state supply during 100*Hz* stimulation (Fig 4B, reciprocal of y-axis intercept of green line; see **Lemma 1** in the **Methods** for details). Indeed, the time course of decay in response size seen after the frequency jumps was clearly slower than the decay during trials where 300*Hz* stimulation was initiated from rest (Fig 4C), as expected if *p*_*v*_ was lower for vesicles remaining in the RRP after partial depletion with 100*Hz* stimulation; this result is in-line with previous frequency jump experiments at the calyx of Held conducted at lower frequencies [29].

### Sequential *vs* parallel models of vesicle priming

The result is not compatible with the simplest models where all RRP vesicles always have the same *p*_*v*_. This was expected because the simplest models were already strongly questioned by the previous evidence that the RRP is subdivided into slow- and fast-releasing subdivisions when synapses are fully rested. Moreover, the result is consistent with the current concept that vesicle priming is sequential, whereby vesicles that are newly recruited to the RRP initially have a low *p*_*v*_[20]; sequential priming models include the concept of positional priming where *p*_*v*_ for newly recruited vesicles increases over time as vesicles that are docked and molecularly primed are translocated to areas of high Ca^2+^-channel density [21]. However, the result is additionally consistent with the fundamentally different alternative where readily releasable vesicles dock to separate sets of release sites with intrinsically low and high *p*_*v*_[9, 23]. We refer to the second possibility as a parallel model because vesicles with low and high *p*_*v*_ would be recruited in parallel. Finally, *p*_*v*_ for a homogeneous population of releasable vesicles might have decreased *a posteriori*, after the start of stimulation, owing to use-dependent fatigue of the release machinery or even inactivation of Ca^2+^-channels [31].

### Terminology: Reluctant *vs* slow-releasing vesicles

Regardless of mechanism, we use the terms “reluctant” and “reluctantly-releasing” to describe readily releasable vesicles with low *p*_*v*_[23, 32]. We do not assume that *p*_*v*_ must be the same for all reluctant vesicles, and in fact we leave open the possibility that newly recruited vesicles go through multiple stages of priming with ascending values for *p*_*v*_, possibly starting from *p*_*v*_ = 0. To maintain terminology that is consistent with previous reports we reserve the term “slow-releasing” to describe the vesicles that are found within the slow-releasing subdivision of the RRP after long periods of rest. Below we show that sequential models predict that the mechanism for low *p*_*v*_ vesicles in the flow-through pool is different than the mechanism for low *p*_*v*_ vesicles in the slow-releasing subdivision of the RRP, whereas parallel models predict that the mechanism is the same (*i.e.*, see **Lemma 7**).

### Synchronous release of reluctant vesicles

**Fig 5:**
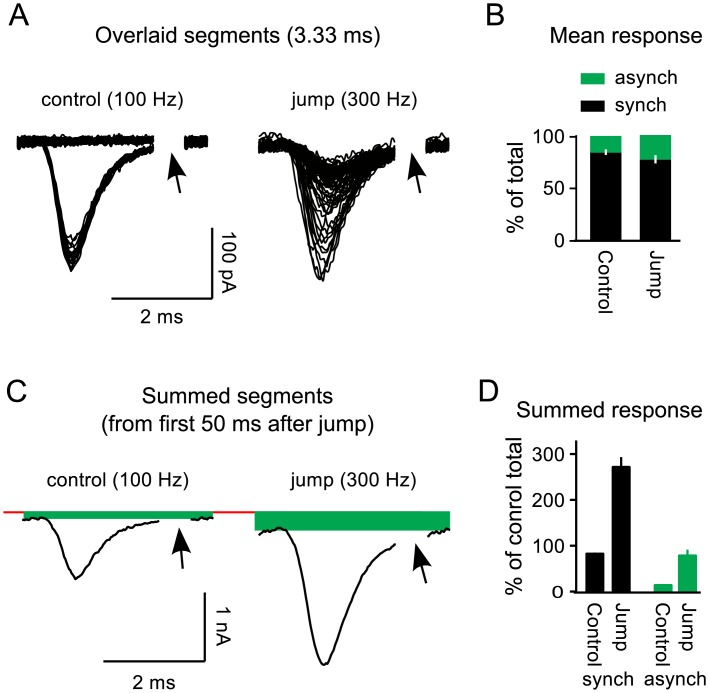
Synchronous release of reluctant vesicles. **A.** Overlaid segments from the entire 200*ms* after the frequency jump and controls where stimulation was 100*Hz* over a matching time window. 23 of segments from control trials (left) only contained asynchronous responses, which are the nearly horizontal traces in the plot. In contrast, all segments after the frequency jump (right) contained an EPSC synchronized to an action potential because stimulation was 300*Hz*. Arrows indicate regions that were removed because they contained residual components of the stimulus artifact when stimulation was 300*Hz*. **B.** Quantification of relative sizes of synchronous and asynchronous components of the mean response over the first 50*ms* at 300*Hz* during the frequency jump trials, and the 100*Hz* controls at matching times. Note that most of the transmitter continued to be released synchronously after the frequency jump (*n* = 7). **C.** Summed segments over the first 50*ms* after the frequency jump and the 100*Hz* controls at matching times. The solid red horizontal line is the baseline calculated over the 100*ms* before stimulation was initiated and the offsets demarcated by green boxes represent the asynchronous component. **D.** Quantification of relative sizes of synchronous and asynchronous components of the summed response showing that much more transmitter was released synchronously after the frequency jumps compared to controls where stimulation frequency was not increased, but was instead maintained at 100*Hz*.

A current concept is that slow- or reluctantly-releasing vesicles are released asynchronously, with a delay or slow kinetics after the triggering action potential, possibly owing to a final priming step taking tens of *ms*[19, 33, 34]. However, we found that the increased release elicited by the frequency jumps was tightly synchronized to action potentials (Fig 5A and 5B).

To quantify the amount of synchronous *vs* asynchronous release, we calculated the integrals of 3.33*ms* segments after removing the asynchronous component, and divided them by the full integral calculated beforehand. The asynchronous component was removed by subtracting a baseline measured between 2.8 and 3.3*ms* after each pulse of stimulation (illustrated in Fig 5C). Only difference traces were analyzed because the stimulus artifacts in the raw data occluded the baseline window (*n* = 7, as noted above).

The measurement would underestimate the synchronous fraction if the time courses of individual responses did not run to completion before the subsequent action potential, which seems likely during 300*Hz* stimulation. Even so, the synchronous fraction declined only a small amount, at most from 84% ± 3% during 100*Hz* stimulation to 77% ± 4% over the first 50*ms* after increasing the stimulation frequency to 300*Hz* (Fig 5B). Meanwhile, the summed response was 2.7 ± 0.2-fold larger (Fig 5C and 5D). Thus, the frequency jumps transiently increased the synchronous component of release approximately 2.5-fold, and more than 75% of the vesicles with low *p*_*v*_were released synchronously.

### General framework for modeling vesicle recruitment to autonomous release sites

**Fig 6:**
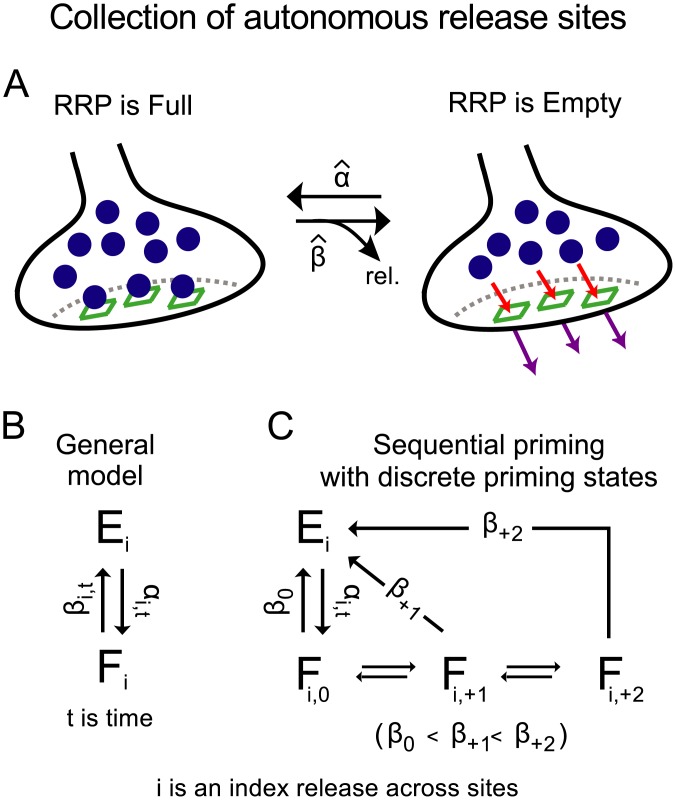
Development of general model described by Eq 1. **A.** RRP conceptualized as a collection of autonomous release sites (green squares). **B.** General model for vesicle recruitment and priming at individual release sites described by Eq 1. Release sites in the F-state (Full) contain a vesicle that is included in the RRP, whereas release sites in E-states (Empty) do not. Recruitment to the RRP at release site *i* is represented by the transition from E_i_ to F_i_. Subsequent priming that increases the probability of release is represented by increasing the value for *β*_*i*_ over time. The general model can describe the behavior of both sequential and parallel models of vesicle priming as explained in the Results. **C.** A more traditional sequential model, also consistent with Eq 1, where vesicle priming progresses through discrete states on the way to full maturity.

The results in Fig 2 argue against the idea that the capacity of the RRP for storing vesicles is a dynamic function of Ca^2+^. In contrast, the results are in-line with the idea that the capacity is constant, which would occur if the RRP were made up of a fixed number of autonomous release sites that could be depleted of vesicles during heavy use (Fig 6A).

The sum of responses during the frequency jump trials was 1.64 ± 0.04-fold larger compared to when stimulation was 300*Hz* throughout, even though both protocols exhausted both fast- and slow-releasing subdivisions of the RRP. A difference of some amount would be predicted by many models that have been proposed, including ones where the RRP has a constant capacity, because some neurotransmitter would have been released from vesicles that were recruited to the RRP during ongoing stimulation, and there was more time for recruitment of new vesicles during the frequency jump trials (700*ms* *vs* 200*ms*)[16, 35]. However, we show below that the standard models employed for estimating the capacity of the RRP and vesicle recruitment rates make predictions that are not quantitatively in line with the result. It was therefore important to test the feasibility of models where release sites are autonomous by comparing the relative amounts of release before and after the frequency jumps and when 300*Hz* stimulation was initiated from rest.

To describe the analysis, we first distinguish between unitary and bulk concepts of recruitment and release. The *unitary rate of vesicle recruitment* is the fraction of vacant space within the RRP that is replenished in a given amount of time. That is, if the RRP is made up of autonomous release sites, the unitary rate would be the rate at which a release site recruits a vesicle and consequently becomes full. In contrast, *bulk recruitment* is the rate at which vesicles are recruited to the RRP as a whole, and is fast at the calyx of Held in part because the RRP is large—*i.e.*, with thousands of release sites—and in part because the unitary rate is faster than at standard synapse types, which is shown below. By definition, the unitary recruitment rate equals the bulk rate divided by the capacity for storing vesicles when the RRP is empty; for Fig 6A the capacity would be the number of release sites. However, the bulk rate is only guaranteed to be linearly related to the unitary rate when the RRP is empty because a key consequence of models such as in Fig 6A is that new vesicle recruitment would be blocked from release sites that were already full.

Likewise, the *unitary rate of release* is the fraction of the vesicles within the RRP that are released in a given unit of time, which is equivalent to *p*_*v*_ multiplied by the stimulation frequency. Meanwhile, the *bulk release rate* is the aggregate rate of release from the entire RRP and is not necessarily related to the unitary release rate in a straightforward way; *e.g.*, at hippocampal synapses, at least, the bulk rate can be depressed owing to RRP depletion at the same time when the unitary rate is enhanced by residual Ca^2+^-dependent mechanisms [11].

A single release site is depicted by the Markov chain in Fig 6B where the site switches between two states, either filled with a vesicle (F-state), or empty (E-state). The unitary recruitment rate is depicted as *α*_*i*, *t*_ (where *i* is an index that identifies each release site and *t* is time). *β*_*i*, *t*_is the unitary rate of release. Although the diagram is simple, the model incorporates enough flexibility to reproduce the behavior of all models, sequential or parallel, that are based on the premise that readily releasable vesicles are docked and primed at a fixed number of autonomous release sites.

That is, allowing *β*_*i*, *t*_ to vary in time provided enough flexibility to account for sequential transitions from low to higher *p*_*v*_ stages of vesicle priming and other mechanisms that affect *p*_*v*_ in both positive and negative directions such as paired-pulse facilitation and Ca^2+^-channel inactivation [11, 36–38]. Allowing *β*_*i*, *t*_ to vary across release sites was necessary for parallel models to account for the slow and fast-releasing subdivisions of the RRP and for sequential models to suppress the assumption that vesicle recruitment and subsequent sequential priming transitions are synchronized across release sites.

Meanwhile, allowing *α*_*i*, *t*_ to vary in time and across release sites allowed for possible activity-dependent acceleration of the vesicle recruitment mechanism [39], and possible heterogeneity across release sites [23]. In any case, merely allowing the value of *α*_*i*, *t*_ to change in time and to vary across release sites would not exclude the special cases where *α*_*i*, *t*_ is constant in time or across release sites (but see below).

We refer to general models, such as in Fig 6B, that can reproduce the behavior of entire categories of specific models, as sparse models. Specific models make predictions that are more precise, but often depend on assumptions that have not been verified. In contrast, sparse models can be used to elucidate boundary conditions that must then apply to a wide range of specific models.

In the present case, release and new vesicle recruitment to the RRP could be modeled as:

$$\begin{array}{c} \frac{dRRP_{t}}{dt}={\hat{\alpha }}_{t}\cdot \left(RRP_{0}-RRP_{t}\right)-{\hat{\beta }}_{t}\cdot RRP_{t} \end{array}$$ (1)

where *RRP*_0_ is the number of release sites, *RRP*_*t*_ is the number that are occupied at time *t*, ${\hat{\alpha }}_{t}$ is the mean value of *α*_*i*, *t*_ for all release sites that are empty, and ${\hat{\beta }}_{t}$ is the mean *β*_*i*, *t*_ for all sites that are full. Eq 1 was derived from the model in Fig 6B, and therefore, any boundary conditions established by the equation would be inherited by all models that satisfy the initial premises; see **Lemma 2** in the Methods for the derivation and confirmation that sequential models are covered even when priming occurs through discrete states, such as in Fig 6C.

The initial goal was to use Eq 1 to divide cumulative responses during train stimulation into two fractions: the response generated by releasing all the transmitter in *RRP*_0_; and the response generated by releasing $\int {\hat{\alpha }}_{t}\cdot \left(RRP_{0}-RRP_{t}\right)$, which is the transmitter recruited during the stimulus train and is referred to below as the cumulative recruitment. The idea is that the response generated by releasing *RRP*_0_ would be the same for all stimulation protocols, and therefore, mismatching estimates for *RRP*_0_ from frequency-jump trials compared to when 300*Hz* stimulation was initiated from rest would rule the model out.

We did not attempt to estimate specific values for ${\hat{\alpha }}_{t}$ or ${\hat{\beta }}_{t}$ *a priori*. Instead, we started with the special case where ${\hat{\alpha }}_{t}$ is constrained to be some constant, referred to below as ${\hat{\alpha }}_{fixed}$; the value of ${\hat{\alpha }}_{fixed}$ was not specified *a priori*, but a unique value was determined by the data (see below). Although recognized beforehand as a potential oversimplification, the special case was a convenient starting point because we showed previously that it can be used to extract unique values for ${\hat{\alpha }}_{fixed}$, ${\hat{\beta }}_{t}$, and *RRP*_0_ from experiments where the RRP is exhausted, or at least when driven into a partially empty steady state [9]. The change from *α*, *β*, *N*, and *n* in the previous study to ${\hat{\alpha }}_{fixed}$, ${\hat{\beta }}_{t}$, *RRP*_0_, and *RRP*_*t*_ here is purely notational and does not alter the mathematical relationships that were derived previously.

The analysis involved finding the unique value for ${\hat{\alpha }}_{fixed}$ where $RRP_{0}=\frac{R_{ss}\cdot \nu }{{\hat{\alpha }}_{fixed}}$ when *R*_*ss*_ is the release *per* action potential at steady state and *ν* is the frequency of action potentials. To accomplish this, we used computer simulations to calculate the predicted amount of bulk recruitment for a range of values for ${\hat{\alpha }}_{fixed}$ (see Methods). We then chose the value where the difference between the cumulative release and cumulative recruitment over the entire train was equal to $\frac{R_{ss}\cdot \nu }{{\hat{\alpha }}_{fixed}}$. The mathematically more precise procedure used in [9] produced the same results, but simulations were used in the present study because they allowed flexibility needed below for modeling activity-dependent acceleration of the vesicle recruitment mechanism.

**Fig 7:**
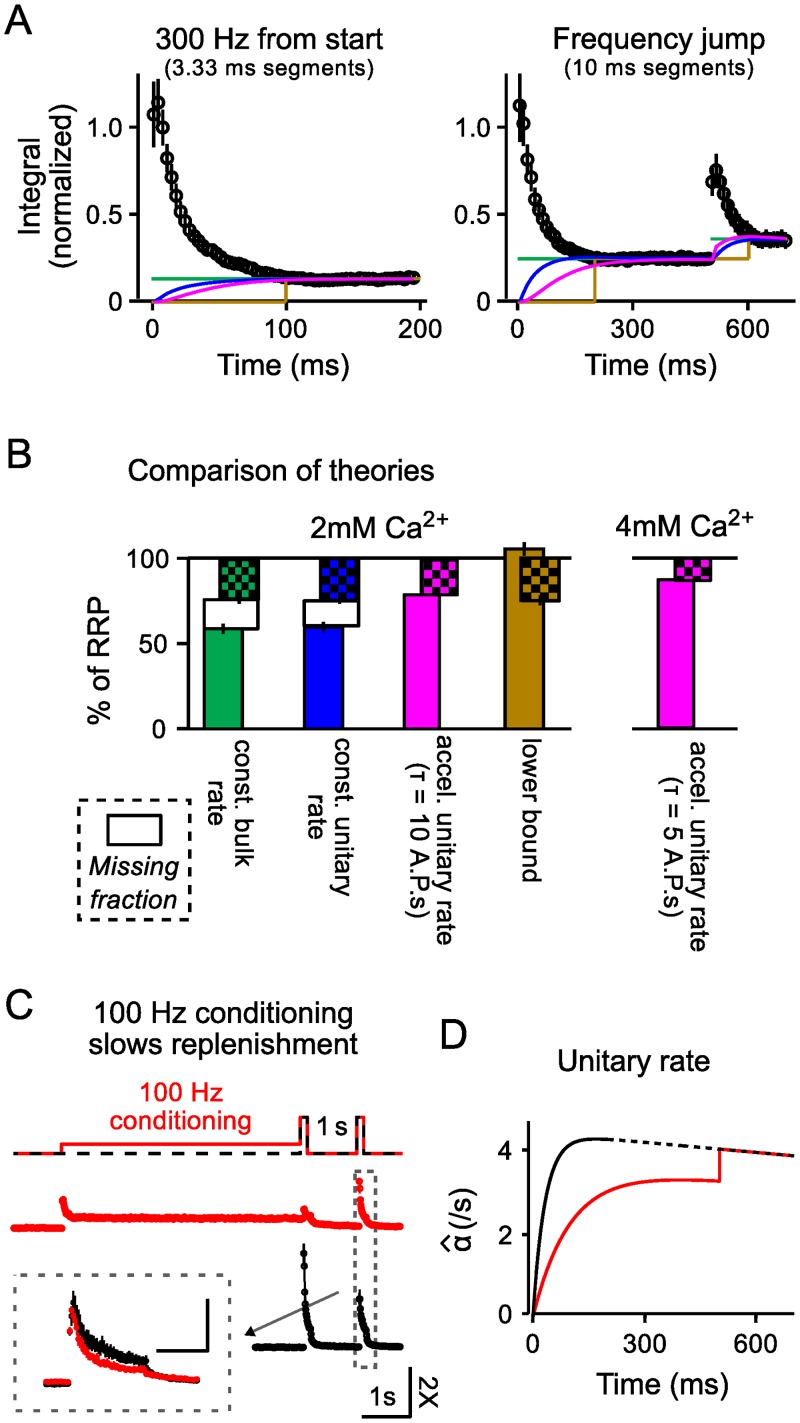
Analysis of vesicle recruitment to the RRP for models covered by Eq 1. **A.** Integral of segments *vs* time during the frequency jump experiments; segments were 3.33*ms* for the 300*Hz* trials (left panel) and 10*ms* for the frequency-jump (right panel). Values are directly comparable to each other because they were identically normalized by the integral of the first 3.33*ms* segment. Colored lines are estimates from a range of models of the response generated by release of vesicles that were recruited to the RRP to replace vesicles expended earlier during stimulation; colors match **Panel (B)**. **B.** Bar graph of estimates of the fraction of the RRP released by 100*Hz* (solid) and subsequent 300*Hz* (checkerboard) stimulation for a variety of models as indicated. **C.** 100*Hz* conditioning slows RRP replenishment over subsequent rest intervals. Top: stimulus protocols. Red represents trials that included 5*s* of 100*Hz* conditioning stimulation, black (dashed) is without prior conditioning. 1*s*-long rest intervals were preceded by 150*ms* of 300*Hz* stimulation for both types of trials to ensure the RRP was empty at the start of the rest interval. Middle and Bottom: Responses from sample traces are plotted as integrals of 10*ms* segments *vs* time. All values were normalized by the integrated trace segment corresponding to the first 3*ms* of stimulation; the 2*X* value on the scale bar signifies 2-times the value used for normalization. Inset: Integrals of segments (3.33*ms*) during 300*Hz* stimulation after the rest interval for both types of trials. Note reduced response after conditioning (red trace). Scale bar in inset is 100*ms* by 0.5-times the value used for normalization. **D.** Estimates of ${\hat{\alpha }}_{t}$ from Eq 1 that were used to incorporate activity-dependent acceleration and later fatigue into models of vesicle recruitment. The black line is the estimate used for trials where stimulation was 300*Hz* throughout and the red is for the frequency jump trials. The exponential constant for acceleration (*τ* ⋅ *ν* in Eq 2) was 10 action potentials for 100*Hz* and 300*Hz* stimulation alike, and fatigue was modeled as a 10%/*s* linear decrease. The dashed section of the black line is an extrapolation that was not tested empirically.

The analysis produced values for ${\hat{\alpha }}_{fixed}$ of 4.65/*s* for the 300*Hz* trains and 4.70/*s* for the frequency jump trials, which are in-line with previous estimates at the calyx of Held [40]. The portion of responses generated by release of newly recruited vesicles for the data plotted in Fig 7A are demarcated by blue lines.

We emphasize that the values for ${\hat{\alpha }}_{fixed}$ pertain specifically to vesicle recruitment to the RRP, not to any subsequent transitions to higher *p*_*v*_ stages of vesicle priming, which would be downstream. Instead, the timing of downstream priming transitions would influence ${\hat{\beta }}_{t}$, and some information could have been extracted at this point in the analysis (*e.g.*, see Figure 2A of [41]). However, interpreting the information in the context of sequential priming would have required making additional assumptions, which we sought to avoid.

In any case, the value of *RRP*_0_ estimated from frequency jump trials was 14 ± 3% less than from trials where 300*Hz* stimulation was initiated from rest (blue bars in Fig 7B), ruling out models covered by the general framework where the unitary rate of vesicle recruitment to the RRP is constant. For comparison to previous studies, the green lines in Fig 7A are from a back extrapolation analysis, which is a standard in the field, that implicitly assumes that the bulk rate of recruitment—as opposed to the unitary rate—is constant throughout [13]; Fig 7B shows that the mismatch was similar for this method (17 ± 3%, green bars), ruling out models where the bulk rate is constant. Finally, ignoring the contribution of recruitment until after achieving a steady state (brown lines in Fig 7A) results in an overestimation of 32 ± 4% (brown bars in Fig 7B), ruling out models where vesicle recruitment does not occur until the RRP is completely empty.

The mismatches between the two estimates for *RRP*_0_ could be eliminated by allowing ${\hat{\alpha }}_{t}$ to accelerate from an initially low value as hypothesized in [39]. This works because acceleration lessens estimates of bulk recruitment during the early part of trains (magenta lines in Fig 7A)—and consequently increases estimates of *RRP*_0_—and the effect is disproportionately larger when the acceleration is induced more slowly, as would be expected during the 100*Hz* trains at the beginning of the frequency jump trials compared to when stimulation was 300*Hz* from the outset.

As proof of the concept that incorporating recruitment rate acceleration could resolve the discrepancy, we modeled the acceleration as the single rising exponential:

$$\begin{array}{c} {\hat{\alpha }}_{t}={\hat{\alpha }}_{max}\cdot \left(1-e^{\frac{-t}{\tau }}\right) \end{array}$$ (2)

where *τ* is a free parameter that could be manipulated to model the time course of engagement of the acceleration mechanism, and ${\hat{\alpha }}_{max}$ is a maximum value that is determined by the data. The idea was that each action potential would increase ${\hat{\alpha }}_{t}$ by a constant fraction up to ${\hat{\alpha }}_{max}$; ${\hat{\alpha }}_{t}$ was already known to approach some maximum because response sizes during 300*Hz* stimulation settle to a steady state and do not increase after the RRP has been exhausted (*e.g.*, Fig 1C), as would occur otherwise [9].

We found that estimates of *RRP*_0_ from the frequency jump trials and trials where 300*Hz* stimulation was initiated from rest were equal when $\tau =\frac{10}{\nu }$, where *ν* is the frequency of stimulation (magenta bars, left panel of Fig 7B). Lower values for *τ* were not sufficient and higher values produced an overcorrection.

More complicated models of the time course of acceleration could resolve the discrepancy equally well. However, a key point is that the acceleration mechanism must increase the rate at which vesicles are recruited to the RRP. No amount of acceleration of downstream transitions to priming stages with higher *p*_*v*_ could substitute. This is notable because some other experimental paradigms and analysis, documented previously, were not able to distinguish clearly between acceleration at the step of recruitment and downstream effects [42]. The distinction is possible in the present case because acceleration of vesicle recruitment is fundamentally different from acceleration of downstream priming steps for models covered by Eq 1 where only vacant release sites recruit new vesicles; release sites occupied by vesicles occlude recruitment, even when *p*_*v*_ = 0.

An analogous analysis of the experiments conducted in 4*mM* Ca^2+^ also required recruitment rate acceleration, but in this case $\tau =\frac{5}{\nu }$, whereas $\tau =\frac{10}{\nu }$ produced an overcorrection (magenta bars, right panel of Fig 7B). The lower value for *τ* indicates that fewer action potentials would be required to accelerate the recruitment mechanism when extracellular Ca^2+^ is higher, which is in-line with the previous studies indicating that recruitment rate acceleration is mediated by Ca^2+^ influx for at least one other synapse type [43].

### Fatigue in recruitment during continuous stimulation

Unexpectedly, when acceleration was incorporated, ${\hat{\alpha }}_{max}$ was fixed by the data to a lesser value for the frequency jump trials than for the trials where 300*Hz* stimulation was initiated from rest (3.9/*s* *vs* 4.3/*s*; the numbers correspond to experiments where Ca^2+^ was 2*mM*). If the modeling framework is correct, the discrepancy would indicate that vesicles were recruited ∼10% more slowly during 300*Hz* stimulation at the end of the frequency jump trials than during the trials where 300*Hz* stimulation was initiated from rest. In other words, the recruitment mechanism must have fatigued a small amount during 500*ms* of 100*Hz* stimulation.

To verify this, we first compared the steady state response size during 300*Hz* stimulation after frequency jumps to the steady state when 300*Hz* stimulation was initiated from rest. The size was 9.9 ± 2.8% lesser for the frequency jump trials, which was predicted by the model because the size of the responses that continue to be evoked when the RRP is exhausted would be proportional to the rate at which new vesicles are recruited [9].

Additional experiments showed that the fatigue persisted long enough to slow RRP replenishment during subsequent rest intervals. We stimulated calyces with pairs of 300*Hz* trains separated by 1*s*-long rest intervals before (black in Fig 7C) and immediately following (red) 100*Hz* conditioning trains lasting 5*s* (see diagram atop Fig 7C). An index of the replenishment occurring over the rest intervals was calculated by dividing the sum of responses during the second 300*Hz* train of each pair by the sum of responses during the first 300*Hz* train of the pair that was initiated without prior 100*Hz* conditioning. The index was 52 ± 5% after 100*Hz* conditioning compared to 76 ± 1% (*n* = 4, *p* < 0.02) without conditioning, which is a decrease of 31 ± 7%. The decrease was more than the 10% measured above likely because 100*Hz* conditioning was 5*s* instead of 500*ms*. Taken together, the identification of fatigue lends support to the modeling framework defined by Eq 1 because the predictions were confirmed with two orthogonal types of experiments that could both be interpreted independently of any model. A related phenomenon may have been identified previously in rats for an earlier developmental stage using a different experimental technique [44].

### Confirmation of activity-dependent acceleration of recruitment to the RRP as a whole

The fatigue in vesicle recruitment could be incorporated into the framework defined by Eq 1 in a variety of ways, but could not be manipulated to eliminate the prediction that activity accelerates the unitary recruitment rate, $\hat{\alpha }$, or even to substantially alter the estimate of $\tau =\frac{10}{\nu }$ when acceleration was modeled with Eq 2 (Fig 7D).

**Fig 8:**
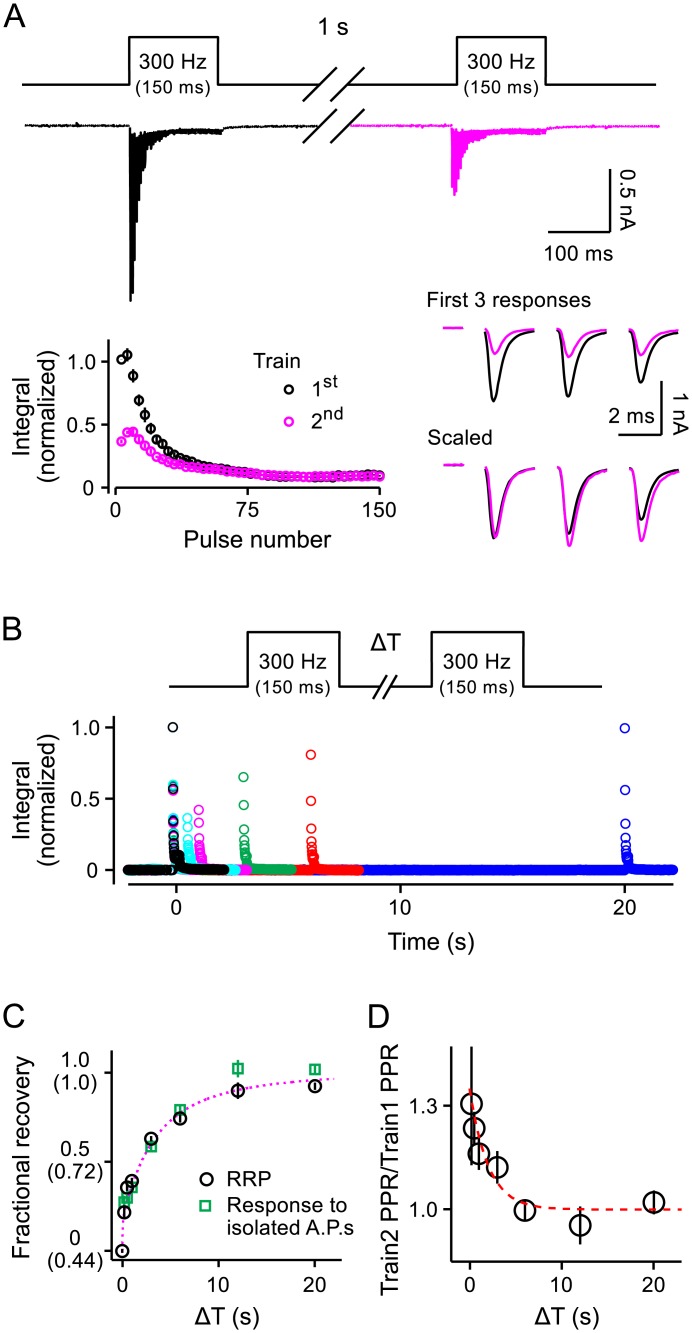
Recovery from depression during rest intervals. **A.** Incomplete RRP replenishment in 1*s*. Stimulation was pairs of 150*ms*-long trains at 300*Hz* separated by 1*s* rest intervals as diagrammed at top. Top trace: Averaged raw data across all experiments after blanking stimulation artifacts; trace is colored magenta during the second train to match the panels below. Bottom left: Quantification; responses were divided into 3.33*ms* segments, integrated, normalized by the value for the first segment, and averaged across trials (black is first train, magenta is second). Bottom right: Overlaid traces from the first 10*ms* of each train illustrate slightly more paired-pulse facilitation during the second train. **B.** Integrated segments (10*ms*) for a series of experiments with a range of inter-train intervals. Data are from a single preparation; multiple traces for each inter-train interval were averaged digitally before segmentation and integration. **C.** Time course of recovery from depression (*n* ≥ 14 trials from 7 preparations). Circles (black) are the integral of the entire second train divided by the integral of the first train. Squares (green) are the integral of the first 3.33*ms* segment of the second train divided by the first segment of the first train. The Y-axis is calibrated so that recovery was 0 when the inter-train interval was 0. The values in parentheses are uncorrected integral of the second train divided by the integral of the first; the value for inter-train intervals of 0 was 0.44, even though the RRP was empty, because of ongoing release of newly recruited neurotransmitter during stimulation. The dashed line (magenta) is Eq 3 in the Results. **D.** Time course of decay of the small enhancement in the paired-pulse ratio caused by prior stimulation. The dashed line is the single exponential $e^{\frac{-t}{\tau }}$ where *τ* = 2*s*.

Although previous studies identified mechanisms that accelerate the recruitment of vesicles from the slow- to fast-releasing subdivisions of the RRP, $\hat{\alpha }$ pertains to the upstream step, where vesicles are initially recruited to the RRP as a whole. To our knowledge, acceleration specifically at the upstream step had not been demonstrated previously for the calyx of Held; the already identified acceleration would instead influence ${\hat{\beta }}_{t}$ in Eq 1. Therefore, to verify the prediction that activity accelerates the rate at which vesicles are recruited to the RRP as a whole, we measured the time course of RRP replenishment during rest intervals that followed action potential trains. We used pairs of 300*Hz* trains separated by experimentally varied rest intervals as diagrammed in Fig 8A and 8B. Each train was 150*ms* at 300*Hz* (45 action potentials) to ensure that both fast and slow-releasing subdivisions of the RRP were exhausted. The fractional amount of RRP replenishment during each rest interval was calculated by dividing the response integral during the entire second train by the integral during the first. Recruitment of vesicles during ongoing stimulation was factored out by including interleaved trials where the rest interval between trains was nominally zero (*i.e.*, 3.33*ms*).

We reasoned that a mechanism that accelerates vesicle recruitment during trains of action potentials would disengage during subsequent rest intervals, causing recruitment to slow down. In contrast, if acceleration did not occur, the unitary recruitment rate would be maintained at a constant during rest intervals, and the RRP would replenish more than 98% during the first 1*s* alone; that is, $RRP_{t}=1-e^{-{\hat{\alpha }}_{max}\cdot t}$ where *RRP*_*t*_ is the fractional fullness of the RRP at time *t* and ${\hat{\alpha }}_{max}=4.3/s$ from above. However, full replenishment took much longer than 1*s* (Fig 8A–8C), supporting the prediction from the general model defined by Eq 1 that the recruitment rate was accelerated by the preceding activity.

At a more quantitative level, the time course of RRP replenishment could not be approximated with any single exponential function (*i.e.*, one with a constant unitary rate); this is in-line with previous measurements [28, 45, 46]. However, the full RRP replenishment time course was closely approximated by:

$$\begin{array}{c} RRP_{t}=1-e^{-\int {\hat{\alpha }}_{t}} \end{array}$$ (3)

where ${\hat{\alpha }}_{t}$ is the decaying double exponential:

$$\begin{array}{c} {\hat{\alpha }}_{t}=\left({\hat{\alpha }}_{max}-{\hat{\alpha }}_{\infty }\right)\cdot \left[w\cdot \left(e^{\frac{-t}{\tau {}_{f}}}\right)+\left(1-w\right)\cdot \left(e^{\frac{-t}{\tau_{s}}}\right)\right]+{\hat{\alpha }}_{\infty } \end{array}$$ (4)

and *w* = 0.95, *τ*_*f*_ = 50*ms*, *τ*_*s*_ = 7*s*, ${\hat{\alpha }}_{max}=4.3/s$, and ${\hat{\alpha }}_{\infty }=\frac{1}{12}/s$ (magenta dashed line in Fig 8C). Eq 3 is relevant because it was derived from Eq 1 by assuming that: ${\hat{\beta }}_{t}=0$, as expected during rest intervals; *RRP*_0_ = 0 because the RRP was empty at the beginning of the rest interval; and *RRP*_*t* → ∞_ = 1.0 because of the way the replenishment values in Fig 8C were normalized [9]; ${\hat{\alpha }}_{\infty }$ would be the baseline unitary recruitment rate expected in the absence of activity.

In this case, Eq 4 would describe the time course over which the acceleration mechanism disengages during rest intervals. If so, disengagement at the calyx of Held would be much faster than at excitatory hippocampal synapses. Nevertheless, the *τ*_*f*_ = 50*ms* value was in-line with expectations because disengagement at excitatory hippocampal synapses followed the clearance of residual Ca^2+^, which is likely much faster at the calyx of Held [47]; the time course of disengagement at hippocampal synapses was measured in [43] and Ca^2+^ clearance in [11].

In sum, the measurements of RRP replenishment during rest intervals and the results from the frequency jump experiments are both in-line with the prediction that activity accelerates the recruitment of vesicles to the RRP [39]. The logic is based on the assumption that the general model defined by Eq 1 is accurate, but further analysis did not yield any alternatives where the requirement for acceleration of the recruitment mechanism could be avoided. Notably, one set of alternatives where vesicles in the slow-releasing subdivision of the RRP are occluded from docking at release sites by vesicles already in the fast-releasing subdivision [48], could account for the results from the frequency jump experiments with a mechanism that accelerates the downstream transition from the slow- to fast-releasing subdivision with no need for accelerating the mechanism that recruits vesicles to the slow-releasing subdivision. However, the results from the RRP replenishment experiments in Fig 8C are not compatible with these alternatives.

That is, further analysis produced model-independent lower and upper bounds for the unitary rate of vesicle recruitment to the RRP of 3.21/*s* ± 0.15/*s* and 4.91/*s* ± 0.37/*s* during 300*Hz* stimulation (brown and green lines in Figs 4A and 7A, respectively). Even the lower bound would predict that the slow-releasing subdivision would replenish more than 96% during the first 1*s* in the absence of a disengaging acceleration mechanism. Meanwhile, we reasoned that the fast-releasing subdivision would have to remain nearly completely empty during the 1*s* interval to account for the <40% RRP replenishment overall because the slow- and fast-releasing subdivisions each make up approximately half of the total [17]. But, an empty fast-releasing subdivision is not compatible with the observations that responses to isolated action potentials recovered as quickly as the RRP as a whole because isolated responses would primarily track replenishment of the fast-releasing subdivision (compare squares to circles in Fig 8C). We did observe a small increase in the paired pulse ratio after short rest intervals (Fig 8D), which is consistent with a transient decrease in the mean value for *p*_*v*_, but even this effect was no longer detectable after 6*s* of rest when RRP replenishment was still far from complete.

Taken together, the results in Fig 8 support the general model because acceleration of the recruitment mechanism is an unavoidable prediction when the general model is applied to the results of the frequency jump experiments, which are orthogonal experiments, but not when alternatives to the general model are applied. Notably, the mechanism that causes this type of acceleration is likely distinct from the calmodulin-dependent and actin-dependent mechanisms that are thought to accelerate downstream steps in sequential priming because blockers largely abolished synchronous release but seem to have a relatively minor impact on the overall rate of recruitment and subsequent release during maximal stimulation [28, 34].

### 115*ms* dwell time in low *p*_*v*_ states for sequential models

Both sequential and parallel models predict that the mean dwell time for readily releasable vesicles would be ∼115*ms* during 100*Hz* trains of action potentials; a longer dwell-time would produce a higher steady state level than was seen (**Lemma 5**). In the context of sequential models, this implies that the unitary rate for traversing the complete set of sequential transitions from the initially low value of *p*_*v*_ to *p*_*v*_ ≫ 6% would be ∼9/*s*. The value is 35-fold faster than estimated previously during rest intervals, but consistent with the evidence that sequential transitions are accelerated by activity *via* mechanisms involving calmodulin, and actin [22, 34].

### Parallel models

On the other hand, parallel models are also fully compatible with the results above. In this case, activity-dependent enhancement mechanisms such as facilitation, augmentation, and post-tetanic potentiation would increase *p*_*v*_, much like sequential priming except the transitions would reverse during rest intervals [3, 11, 37, 38, 49]. The requirement for activity-dependent acceleration of ${\hat{\alpha }}_{t}$ established above would continue to apply; in particular, the requirement could not be explained by disproportionately faster recruitment to release sites with low *p*_*v*_ (**Lemma 6**).

Indeed, parallel models were more parsimonious because of a hard requirement from two orthogonal sets of experiments that low and high *p*_*v*_ release sites would be present in near equal proportions both when the RRP is nearly exhausted and when full. That is, the combination of the size of the steady state supply during 100*Hz* stimulation and the timing of recruitment of vesicles to the RRP extracted from the frequency jump experiments in the present study forces parallel models to predict that release sites with low and high *p*_*v*_ are present in near equal proportions (**Lemma 7**); this would be the average for all calyces, see below for evidence of variation between preparations. The prediction matches direct measurements of the slow- *vs* fast-releasing subdivisions of the RRP when the RRP is full [17]. The accurate prediction based solely on results from the frequency-jump experiments is remarkable because it is extrapolated from the steady state fullness of the RRP when ∼80% depleted, and is independent of the details of short-term depression induced by action potential trains that were initiated when the RRP was full. An incorrect prediction would have ruled out parallel models, whereas sequential models must have at least one additional degree of freedom which could be tweaked to maintain compatibility with a broad range of possible outcomes.

### Global fatigue of the release machinery cannot explain slow-releasing vesicles

Independently of whether vesicles with high *p*_*v*_ are primed sequentially from a state with low *p*_*v*_, or in parallel at a separate type of release site, the framework defined by Eq 1 could be used to derive the mean value for *p*_*v*_ when the level of RRP fullness was in a steady state during 100*Hz* trains (**Lemma 3**). This value could then be combined with information about ${\hat{\alpha }}_{t}$ to confirm the simultaneous presence of vesicles with a variety of values for *p*_*v*_ (**Lemma 8**). The concept of heterogeneity in *p*_*v*_ is already widely accepted, but the evidence that the heterogeneity occurs at a specific point in time is new and notable because it argues against the special case of parallel models where the slow component of release seen during maximal stimulation results from fatigue in the release machinery instead of from depletion of a slow-releasing subdivision of the RRP; these have been termed *a posteriori* models in [31].

### Correlations between steady state supply and short-term plasticity

**Fig 9:**
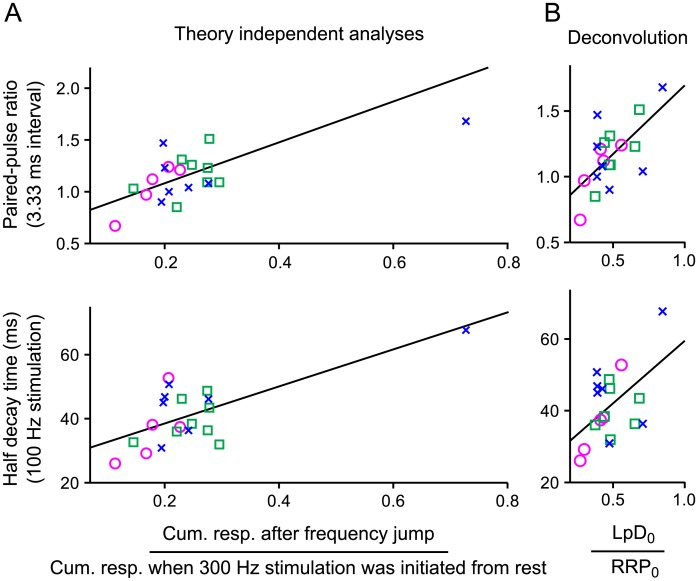
Correlations between steady state supply of reluctant vesicles and short-term plasticity during submaximal stimulation. Data are color coded by age: magenta circles are from animals aged 14–15 days; green squares were 16–18 days; and blue x’s were 19–21 days. **A.** Steady state unreleased fraction of the RRP remaining after 100*Hz* stimulation *vs* the paired-pulse ratio when synapses were rested (top panel) and *vs* the half-decay time for responses during the preceding 100*Hz* stimulation (bottom panel). The steady state fraction was calculated without theory by dividing the cumulative response after the frequency jump by the cumulative response during trials where stimulation was 300*Hz* from the start. Half-decay time was estimated as the 50% point in the cumulative response when stimulation was 100*Hz* from the start. In both cases, cumulative responses were calculated after subtracting the steady state (green lines in Fig 7A); calculating the steady state unreleased fraction instead as the index generated for Fig 3D yielded a similar result. Straight lines are best fits; *p* < 0.001 for both panels. **B.** Capacity of the low *p*_*v*_ subdivision of the RRP assuming the parallel model of vesicle recruitment *vs* the same measures of short-term plasticity used in **Panel (A)**. The procedure factors out variation in new vesicle recruitment and release (see **Lemma 7**; upper bound for ${\hat{\alpha }}_{LpD,ss100}$ was used; one experiment was excluded because the recording quality was too low to estimate the steady state response size, which is required for calculating ${\hat{\alpha }}_{LpD,ss100}$; *p* < 0.01 for both correlations; *R*^2^ = 64% for the top panel, and 50% for the lower).

Finally, results were highly reproducible when trials were repeated on the same preparation, but we observed striking variation between preparations in the size of the steady state supply during 100*Hz* trains compared to the size of the RRP when full. And, the paired pulse ratio at the beginning of trains was greater for preparations which later had larger steady state supplies (Fig 9A, top), and the induction of depression was slower (Fig 9A, bottom). The correlations pertained equally to 100*Hz* and 300*Hz* trains of action potentials (2 of 4 plots are not shown), and were not dependent on age within the 14–21 day range used here (Fig 9).

Variation among calyces in the induction of depression has been reported previously [50], but the correlation with the size of the steady state supply is new. The correlation suggests that the steady state level is mechanistically related to the size of the slowly-releasing subdivision when the RRP is completely full. Meanwhile, a frequent assumption is that low *p*_*v*_ correlates with paired-pulse facilitation and high *p*_*v*_ with paired-pulse depression [11, 50–52], which suggests that the slow-releasing vesicles found in resting RRPs are immediately releasable (see Discussion).

Sequential and parallel models produce fundamentally different explanations for the variation in the steady state level. For sequential models, the steady state would be a function of the timing of transitions from low to high *p*_*v*_ (**Lemma 5**). In contrast, for parallel models, the steady state would be a function of the number of release sites with intrinsically low *p*_*v*_ ($\frac{LpD_{0}}{RRP_{0}}$ in **Lemma 7**). For both parallel and sequential models, the steady state level would additionally be a function of the precise value of *p*_*v*_ for reluctantly-releasing vesicles (**Lemma 3**) and the unitary rate of recruitment to the RRP; the relevant parameter would be ${\hat{\alpha }}_{SS100}$ in **Lemma 4** for sequential models and ${\hat{\alpha }}_{LpD,SS100}$ in **Lemma 7** for parallel models.

Intriguingly, for parallel models, the correlations in Fig 9A translated to correlations between $\frac{LpD_{0}}{RRP_{0}}$ and the same measurements of short-term plasticity (Fig 9B). In contrast, no equivalent correlations emerged for any of the three parameters noted above when the deconvolution was performed assuming sequential priming; the information was likely distributed between ${\hat{\alpha }}_{SS100}$ and the timing of the sequential transitions. The result is an additional indication that parallel models are more parsimonious, and suggests that long-term modulation of the numbers of release sites with intrinsic low *vs* high *p*_*v*_ properties may be a significant determinant of short-term plasticity under a wide range of situations.

## Discussion

The present study yielded new findings specific to the timing of synaptic vesicle trafficking at calyces of Held that are discussed below. A more general point is that we have now shown that the RRP at two very different types of synapses can be well-defined, in the mathematical sense, in a way that is very much in-line with the original concept proposed 50 years ago for neuromuscular junctions [1].

That is, the utility of the RRP concept for quantitative analysis has been questioned [12, 15]. However, here we show that a general kinetic model with minimal assumptions that was employed previously at Schaffer collateral synapses applies equally well to calyces of Held. Despite enough flexibility to cover most current ideas, the model is specific enough to calculate the timing of vesicle recruitment to the RRP with no free parameters, and to confirm a previously uncertain requirement at the calyx of Held for activity-dependent acceleration of the underlying mechanism by which vesicles are recruited to the RRP as a whole (see below).

Assumptions required for other modeling approaches pertaining to the probability of release or rate of vesicle recruitment to the RRP are avoided by the general model [16]. Instead, the premises are only that: (1) the RRP can be exhausted by driving transmitter release heavily; (2) that the capacity for storing vesicles does not change dynamically; and (3) that the rate of vesicle recruitment is a function of the amount of empty space within the RRP. The first two premises are supported by the finding in Fig 2 that the cumulative release during 300*Hz* stimulation is constant when *p*_*v*_ is increased by increasing extracellular Ca^2+^ levels, and the finding that the total number of quanta matches the total released ∼10-fold more quickly by massive increases in intracellular Ca^2+^[17]. The first two premises have additionally been verified for a different type of synapse with orthogonal techniques in cell culture where osmotic shocks and action potentials were used in parallel to exhaust the RRP in the presence and absence of Ca^2+^ within the presynaptic terminals [7, 8, 10].

The third premise was introduced to match the intuitively appealing concept that the RRP is made up of a fixed-number of autonomous release sites [4], and is further supported by analysis and experiments in Figs 7 and 8. That is, the third premise combined with the information from the frequency jump experiments forces the twin predictions of activity-dependent acceleration of the vesicle recruitment to the RRP as a whole during the first tens of action potentials, and later, during continued trains, of a much smaller amount of fatigue in the same mechanism. The two predictions are then confirmed in Figs 8C and 7C, respectively, by experiments that do not depend on any particular theory. Together, the twin confirmations justify adding the third premise independently of the intuitive appeal. However, the results do not prove the separate conjecture that the RRP is made up of a stable population of autonomous release sites, because it would be possible to develop alternative physical interpretations of the model [23, 53].

### Sequential *vs* parallel models

The current concept is that newly recruited RRP vesicles either are not releasable initially, or are only releasable with low *p*_*v*_; *p*_*v*_ is then thought to increase over time as the state of priming matures. These are termed sequential priming models, which include: models where the molecular machinery for catalyzing exocytosis assembles gradually over time; and positional priming models where the rate limiting step is instead translocation to Ca^2+^-channels [20, 21]. However, parallel models where vesicles with low and high *p*_*v*_ are recruited to distinct types of release sites remain possible.

Notably, as long as the fixed capacity principle is retained, all parallel and most sequential models are covered by the general model, including positional priming models where the capacity of a fast-releasing subdivision of the RRP is determined by limited availability of domains with high numbers of Ca^2+^ channels [21]. In contrast, some sequential models where fast-releasing vesicles occlude the transition of slow-releasing vesicles to a dedicated fast-releasing subdivision of the RRP are technically not covered, although in most cases these nevertheless behave like models that are covered when action potential frequency is high enough to exhaust the fast-releasing subdivision.

Sequential and parallel models both predict that the RRP will transform into a flow-through pool containing vesicles with low *p*_*v*_ during submaximal stimulation, which is in-line with the steady state supply identified here during 100*Hz* stimulation. Parallel models are more parsimonious because they reference a single mechanism to explain the presence of slow-releasing vesicles when the RRP is full and vesicles with low *p*_*v*_ when the RRP is ∼80% depleted. In contrast, sequential models require an additional mechanism that could be spontaneous reverse transitions from high back to low *p*_*v*_ states, as in [47], or a limiting number of Ca^2+^ channels [21]. Whatever the identity, variation between preparations in the mechanism that establishes a slow-releasing subdivision within the RRP during rest intervals would have to correlate with variation in the mechanisms that determine the size of the flow-through pool to account for the results in Fig 9A.

There is evidence suggesting that at least some of the heterogeneity between vesicles with low and high *p*_*v*_ arises from intrinsic differences in release sites rather than a variety of stages in sequential priming, which is in-line with parallel models [54–56]. Nevertheless, the evidence for sequential priming is also intriguing [21, 22, 34]. Merging parallel and sequential ideas is possible, but only in a way that would make the sequential transition from not releasable to *p*_*v*_ ≫ 6% faster than 9/*s*, which is already 35-fold faster than estimates from resting calyces of Held [22].

### Models for computational neuroscience

Models based on [57] that have been used widely to implement short-term depression in neural network simulations would be covered by the general model, but are not compatible with the present results because *p*_*v*_ is forced to be the same for all readily releasable vesicles; this limitation was already present in the earliest models for neuromuscular junctions [1, 4]. Other similar computational models, such as in [58], are not strictly compatible with the premise of a constant capacity RRP because depletion does not occur more quickly after the induction of short-term enhancement, but subsequent studies have suggested that enhancement mechanisms increase *p*_*v*_ and therefore speed depletion [8, 37, 38].

### Acceleration of the initial recruitment step

Activity and Ca^2+^-dependent acceleration of synaptic vesicle trafficking has been investigated previously at a broad range of synapse types [43, 59, 60], including the calyx of Held [39] where some of the molecular biology and pharmacology is already known [22, 28, 34, 39, 46]. However, the standard experimental techniques do not automatically distinguish between acceleration of the mechanism by which vesicles are initially recruited to the RRP as a whole and acceleration of downstream mechanisms, such as sequential transitions from a slow- to fast-releasing subdivision [11, 16, 42]. Indeed, the calmodulin and actin-dependent mechanisms that have received most of the attention at the calyx of Held seem to be downstream mechanisms [22, 28, 34]. However, to our knowledge, information about whether or not activity additionally accelerates the upstream step where vesicles are initially recruited to the RRP was only previously available for excitatory hippocampal synapses [42, 43]. Nevertheless, we show here that the initial recruitment step is accelerated many fold by activity as originally predicted [39]; the molecular mechanism remains to be identified, but presumably does not involve calmodulin because inhibitors of calmodulin dramatically alter the kinetics of release but do not effect much recruitment to the slow-releasing RRP subdivision [34].

### Comparison to Schaffer collateral synapses

The emerging conceptual similarities between the calyx of Held and Schaffer collateral synapses are remarkable, but we did find notable differences in parameter values. (1) Recruitment of new vesicles to the RRP during maximal use was ∼15-fold faster at the calyx of Held (4.3/*s* here *vs* 0.24/*s* at matching temperature in [9]). (2) Disengagement of the acceleration mechanism during rest intervals was two orders of magnitude faster (50*ms* here *vs* 10*s* in [43]). And (3) mean *p*_*v*_ for all vesicles in the RRP when rested was higher (11.8 ± 1.2%—*n* = 20—*vs* 4.4% in [9]). In fact, mean *p*_*v*_ for the vesicles with low *p*_*v*_ was similar to the mean for all vesicles in rested RRPs at Schaffer collateral synapses (see **Lemma 3**), whereas *p*_*v*_ for the vesicles with high *p*_*v*_ was ∼5-fold higher (**Lemma 9**).

The conceptual similarities make sense because the molecules are similar [2]. Indeed, the much faster disengagement of the acceleration mechanism at the calyx of Held could simply reflect much faster clearance of residual Ca^2+^ (compare [47] to [11]). In contrast, the faster recruitment of vesicles to the RRP during maximal use likely indicates a difference in the molecular mechanism itself. Intriguingly, the time course of RRP replenishment during rest intervals was not largely different at the calyx of Held compared to Schaffer collateral synapses [9], consistent with the possibility that the difference at the level of molecules is a single player involved in implementing the acceleration of vesicle recruitment to the RRP. In any case, the absence of fast vesicle recruitment during heavy use at Schaffer collateral synapses, and during rest intervals at both types of synapse, does not appear to be an intrinsic limitation of the biological material, suggesting instead a physiological role that remains to be elucidated [40, 61–63].

### Single action potential pools and an effective RRP

There is no contradiction between the premise that the RRP has a fixed capacity and the concept that only a small subdivision might be immediately or effectively releasable at any given time [15, 52]. However, the concept implies that some readily releasable vesicles are not immediately or effectively releasable, which is contrary to the original idea that RRP vesicles are ready to release. And, correlations between RRP size and responses to isolated action potentials [51, 53], and the correlations between the paired pulse ratio, time course of induction of depression, and size of the steady state supply of vesicles during 100*Hz* trains of action potentials in Fig 9, all suggest that even vesicles within the slow-releasing subdivision of the RRP are immediately releasable, although with a low value for *p*_*v*_. In any case, even if some vesicles within the RRP are not immediately releasable, the transition to a releasable state can occur rapidly, in less than 10*ms*, when Ca^2+^ influx is massive [17].

### High and low pass frequency filters

Vesicles with high *p*_*v*_ can be thought of as low pass frequency filters of information encoded by spike trains, whereas vesicles with low *p*_*v*_ are high pass filters [23]. This potential source of computational power is often neglected in models of neural networks, partly because the principles of operation have not been clear. One key issue is whether vesicles with low and high *p*_*v*_ can co-exist in RRPs of synapses with single active zones. That is, the release sites in mathematical models are likely the functional correlate of morphological docking sites in active zones, and it is possible that all vesicles docked at any given active zone all have similar values for *p*_*v*_; *e.g.*, owing to the density of Ca^2+^-channels [64, 65]. This is an important topic because the synaptic connection between pairs of neurons in hippocampus and in other brain regions is often *via* a single synapse containing only one active zone [66]. Thus, determining if synapses with single active zones can simultaneously process vesicles with low and high *p*_*v*_—allowing multiple types of frequency filtering—will be important for understanding computational principles in circuits throughout the brain.

## Methods

### Ethical approval

Animal protocols were approved by the University of Nevada and Universidad de Navarra and conformed to the guidelines of the National Institutes of Health and Spanish Royal Decree 1201/2005.

### Preparation

Tissue from *n* = 32 animals of both sexes was used for this *in vitro* study. Animals were rapidly decapitated, without anesthesia. Transverse slices (200*μm*) containing the MNTB were prepared from C57/Bl6 mice (14–21 days old) in ice cold dissection solution as previously described [67], except the dissection solution was (in *mM*): 85 NaCl, 2.5 KCl, 25 NaHCO_3_, 1.25 NaH_2_PO_4_, 25 glucose, 75 sucrose, 7 MgCl_2_, 0.5 CaCl_2_, 0.4 L-ascorbic acid, 3 myo-inositol, and 2 Na-pyruvate. After cutting, slices were incubated in artificial cerebrospinal fluid (ACSF) for 45–60*min* at 35C, and subsequently stored at room temperature (22–24C) for up to 6 hours. ACSF was (in *mM*): 125 NaCl, 2.5 KCl, 2 CaCl_2_, 2 MgCl_2_, 25 glucose, 25 NaHCO_3_, 1.25 NaH_2_PO_4_, 0.4 ascorbic acid, 3 myo-inositol, and 2 Na-pyruvate. Both solutions were continuously oxygenated with a gas mixture of 95% O_2_/5% CO_2_.

### Recordings

Slices were bathed in a <1*ml* recording chamber with ACSF that was continuously refreshed at approximately 2*ml*/*min*. When used, KYN was added in powder form to already oxygenated ACSF and stirred vigorously for at least 0.5 hours prior to use. Neurons were visualized with infrared differential interference contrast microscopy (BX51WI, Olympus, Japan) *via* a 40x water-immersion objective. Axons from the ventral cochlear nucleus were stimulated with a bipolar tungsten electrode spanning the ventral stria at the mid-line, and excitatory post synaptic current (EPSCs) responses were recorded from principal MNTB neurons, voltage clamped at −70*mV* with a PC-505B amplifier (Warner Instruments, USA), or a Multiclamp 700B (Axon).

Stimulus intensity was set ∼25% above threshold and was 2–3.5*V* for 50–100*μs*. AMPA-receptor mediated EPSCs were isolated with 100*μM* DL-APV, 50*μM* picrotoxin, and 0.5*μM* strychnine. Intracellular recording solution was (in *mM*): 130 Cs-gluconate, 10 CsCl, 5 Na_2_ phosphocreatine, 10 HEPES, 5 EGTA, 10 TEA-Cl, 4 MgATP, and 0.3 GTP, with pH adjusted to 7.2. Recording pipettes were pulled from thick-walled borosilicate glass (*GC*150*F* −10, Harvard Apparatus, USA) with a Sutter P-97 electrode puller to open tip resistances of 1.6–2.5*MΩ*. Series resistance in whole-cell recording configuration was <10*MΩ*, and was compensated 80–92%. All recordings were at room temperature. For most recordings, amplifier, stimulation, and data acquisition were controlled by a computer running in-house software on top of a Debian Linux operating system patched for real-time functionality with the RealTime Application Interface for Linux (www.rtai.org); the data for experiments in S1 Fig were recorded using PClamp.

### Experimental design and analysis

It was often possible to repeat several trials of each experiment in individual preparations and the digitized recordings of identical trials were averaged before further analysis. Preparations were always allowed at least 60*s* of rest before each experiment was initiated to allow synapses to recover completely between trials. For the experiments with a single experimental variable, the experimental and control trials were alternated. For time courses, the order of trials was shuffled. A minimum of 5*min* was allowed for solution exchange when adding drugs or changing the Ca^2+^ concentration. Analysis was accomplished using in-house software written in C++ and MATLAB.

### Statistics

Aggregate data in figures and text are summarized with mean ± s.e.m. Statistical significance from pairwise comparisons was assessed with the Kolmogorov-Smirnov test: * = *p* < 0.05; ** = *p* < 0.01; *** = *p* < 0.001.

### MATLAB code for estimating ${\hat{\alpha }}_{t}\cdot \left(RRP_{0}-RRP_{t}\right)$ from Eq 1 and data

**function** RecruitVals = SimulateRecruit(Resps, AlphaLUT, SegLen)

 Vacancy(**length**(Resps)) = 0;

 RecruitVals(**length**(Resps)) = 0;

 **for** i = 2:(**length**(Resps))

  Vacancy(i) = Vacancy(i − 1) + Resps(i − 1) − RecruitVals(i − 1);

  % *Vacancy is RRP(0) − RRP(t)*

  RecruitVals(i) = Vacancy(i)*LookUp(i − 1, SegLen, AlphaLUT)*SegLen;

 **end**;

**function** alpha = LookUp(RespIndex, SegLen, LUTable)

 time = (RespIndex—0.5)* SegLen;

 [ ˜, ClosestIndex] = **min**(**abs**(LUTable(:,1) − time));

 alpha = LUTable(ClosestIndex(1),2);

### Notes for MATLAB code

1. Resps is a vector of response sizes during a train of stimulation. The precondition is that the fullness of the RRP has reached a steady state level by the end of the train; the steady state level is often close to empty, but this is not required.
2. AlphaLUT is a matrix with 2 columns (time (*s*) and unitary rate (/*s*))
3. SegLen is the length of the segments corresponding to individual responses (units in *s*)
4. RecruitVals is the blue line in Fig 7A when Resps is the vector of response sizes plotted in the same figure and AlphaLUT is the single row [0 4.65] (left panel) and [0 4.70] (right panel). The AlphaLUT tables plotted in Fig 7D generate RecruitVals vectors that are not distinguishable by eye from the magenta lines in Fig 7A, although the lines that are plotted were generated with AlphaLUTs that did not include a factor for fatigue, as described in **Results**.

#### Lemma 1 *Boundary conditions for p*_*v*_ *when RRP is full and during* 100*Hz* *stimulation*

The mean value for *p*_*v*_ when the RRP is full equals the size of the first response during a train of stimulation divided by the aggregate response generated by releasing the entire RRP. However, estimates of the aggregate response generated by releasing the entire RRP depend on the assumptions about the timing of vesicle recruitment, making the precise values of estimates of *p*_*v*_ a function of the same assumptions [9, 68]. The cumulative response during 300*Hz* stimulation would be an overestimate of the aggregate because it would include responses generated by transmitter released from vesicles that were recruited after stimulation was initiated. Therefore, a lower bound for the mean value of *p*_*v*_ could be calculated by dividing the size of the first response by the cumulative response during 300*Hz* stimulation, and was 0.097 ± 0.010 (Fig 4A, brown line). Conversely, an upper bound of 0.14 ± 0.013 could be calculated by assuming that the bulk rate of recruitment is maximal from the start (Fig 4A, dotted green line; see the **Results** section for definition of bulk rate).

The same strategy could be applied to the increase in responses elicited by the frequency jump in order to calculate an upper bound for *p*_*v*_ of 0.064 ± 0.004 that pertains specifically to the steady state supply seen during 100*Hz* stimulation (Fig 4B, green line); the corresponding lower bound was 0.040 ± 0.002 (Fig 4B, dotted brown line).

The steady state supply is interpreted as a flow-through pool (see Results). A key point is that the lower bound for the mean *p*_*v*_ when the RRP was full was higher than the upper bound for the steady state supply. This demonstrates that the mean value for *p*_*v*_ for the vesicles within the flow-through pool was lower than the mean for all vesicles contained in the RRP at the start of stimulation. The lower value for *p*_*v*_ would predict that the standing steady state supply would be depleted more slowly during 300*Hz* stimulation than the RRP when full, which is in line with the slower induction of depression (Fig 4C).

#### Lemma 2 Derivation of Eq 1 and ${\hat{\alpha }}_{t}$ **and** ${\hat{\beta }}_{t}$ from Fig 6B

Each release site *i*, at time *t*, can be either full (*i.e.*, in the F-state):

$$\begin{array}{c} \left\{\begin{array}{c} F_{i,t}=1,\;E_{i,t}=0 \end{array}\right\} \end{array}$$

or empty (E-state):

$$\begin{array}{c} \left\{\begin{array}{c} F_{i,t}=0,\;E_{i,t}=1 \end{array}\right\} \end{array}$$

If the RRP has *RRP*_0_ release sites, then the RRP contents at time *t* is:

$$\begin{array}{c} RRP_{t}=\sum_{i=1}^{RRP_{0}}F_{i,t} \end{array}$$

and

$$\begin{array}{c} RRP_{0}-RRP_{t}=\sum_{i=1}^{RRP_{0}}E_{i,t} \end{array}$$

Each release site can have a unique value for *α* (new vesicle recruitment) and *β* (release) at each time, denoted by *α*_*i*,*t*_ and *β*_*i*,*t*_. Since only sites in the F-state release transmitter, and only sites in the E-state recruit replacement vesicles:

$$\begin{array}{c} \frac{dRRP_{t}}{dt}=\sum_{i=1}^{RRP_{0}}\left(\alpha_{i,t}\cdot E_{i,t}\right)-\sum_{i=1}^{RRP_{0}}\left(\beta_{i,t}\cdot F_{i,t}\right) \end{array}$$

which reduces to Eq 1 when:

$$\begin{array}{c} {\hat{\alpha }}_{t}=\frac{\sum_{i=1}^{RRP_{0}}\left(\alpha_{i,t}\cdot E_{i,t}\right)}{\sum_{i=1}^{RRP_{0}}E_{i,t}} \end{array}$$

and:

$$\begin{array}{c} {\hat{\beta }}_{t}=\frac{\sum_{i=1}^{RRP_{0}}\left(\beta_{i,t}\cdot F_{i,t}\right)}{\sum_{i=1}^{RRP_{0}}F_{i,t}} \end{array}$$

The model in Fig 6C is also covered by Eq 1. By similar logic as above, ${\hat{\alpha }}_{t}$ is the same, whereas:

$$\begin{array}{c} {\hat{\beta }}_{t}=\frac{\sum_{i=1}^{RRP_{0}}\sum_{j=\{0,+1,+2\}}\left(\beta_{j}\cdot F_{i,j,t}\right)}{\sum_{i=1}^{RRP_{0}}\sum_{j=\{0,+1,+2\}}F_{i,j,t}} \end{array}$$

Although more traditional, this method of modeling sequential priming is less general than in Fig 6B because priming stages occur in discrete steps, and cannot be continuously graded. In addition, there is no mechanism for modeling enhancement mechanisms, such as facilitation, that reverse during rest intervals (this could be fixed by allowing *β* values to vary continuously in time as in Fig 6B).

#### Lemma 3 Mean *p*_*v*_ = 4.7% for steady state supply during 100*Hz* stimulation

A more precise estimate of the mean value of *p*_*v*_ for the vesicles within the steady state population during 100*Hz* stimulation could be obtained by using the general framework defined by Eq 1. This is calculated simply as:

$$\begin{array}{c} p_{v}=\frac{R_{ss}}{RRP_{SS100}}=5.5\;\pm \;0.4\;\%\;\left(n=20\right) \end{array}$$

where *R*_*ss*_ is the release *per* action potential at steady state and *RRP*_*SS*100_ is the checkerboard magenta bar in Fig 7B, except in the same units (*pC*) as *R*_*ss*_. The value obtained by analyzing the entire data set after digitally averaging the raw traces was slightly lower; *i.e.*, *p*_*v*_ = 4.7%—the lower value is used below in **Lemmas 5, 7, and 8**.

#### Lemma 4 ${\hat{\beta }}_{SS100}=13.5/s$

When the RRP is in a steady state, such as during 100*Hz* stimulation, $\frac{dRRP_{t}}{dt}=0$. Therefore, from Eq 1:

$$\begin{array}{c} {\hat{\beta }}_{SS100}={\hat{\alpha }}_{SS100}\cdot \left(\frac{RRP_{0}}{RRP_{SS100}}-1\right) \end{array}$$ (5)

where ${\hat{\beta }}_{SS100}$ and ${\hat{\alpha }}_{SS100}$ are ${\hat{\beta }}_{t}$ and ${\hat{\alpha }}_{t}$ for times when the RRP is in a steady state.

$$\begin{array}{c} {\hat{\alpha }}_{SS100}=\frac{R_{ss}\cdot \nu }{RRP_{0}-RRP_{SS100}}=3.6/s \end{array}$$

where *R*_*ss*_ is the release *per* action potential at steady state, *ν* = 100/*s* is the frequency of action potentials, and *RRP*_0_ − *RRP*_*SS*100_ is the solid magenta bar in Fig 7B, except in the same units (*pC*) as *R*_*ss*_. Also from Fig 7B:

$$\begin{array}{c} \frac{RRP_{SS100}}{RRP_{0}}=0.21,\text{and thus}\frac{RRP_{0}}{RRP_{SS100}}-1=3.76 \end{array}$$

Combining these values with Eq 5 yields:

$$\begin{array}{c} {\hat{\beta }}_{SS100}=13.5/s \end{array}$$ (6)

The 13.5/*s* value was obtained by analyzing averaged raw data from *n* = 20 preparations and is used below in **Lemma 5**. The mean value when experiments were analyzed individually was 12.1/*s* ± 1.0/*s*.

#### Lemma 5 Sequential models: Lower bound of sequential transitions during 100*Hz* stimulation

For sequential models, vesicles with low *p*_*v*_ undergo exocytosis by either of two pathways: (1) directly; or (2) after sequential priming to a stage where *p*_*v*_ is higher. From **Lemma 3**, the mean value for *p*_*v*_ for the vesicles within the steady state supply was 4.7%, implying that the vesicles underwent exocytosis directly at an average unitary rate of 4.7/*s*; *i.e.*, because action potentials occurred at 100/*s*. Since ${\hat{\beta }}_{SS100}=13.5/s$ from **Lemma 3**, sequential priming followed by exocytosis would have to be: ${\hat{\beta }}_{SS100}-4.7/s=8.8/s$.

The 8.8/*s* value pertains to the sequential priming step or steps followed by the exocytosis step, and would therefore be a lower bound for sequential priming because exocytosis itself would not be instantaneous, but would depend on the value or values of *p*_*v*_ at more advanced stages of priming. The 8.8/*s* value implies a surprisingly short dwell time of less than $\frac{1s}{8.8}$ or 114*ms* that pertains specifically to vesicles with low *p*_*v*_ that transition on to more advanced stages of vesicle priming before undergoing exocytosis; the mean dwell time for all vesicles with low *p*_*v*_ would be less during 100*Hz* stimulation (*i*.*e*., 74*ms*, which is $\frac{1}{{\hat{\beta }}_{SS100}}$) because some low *p*_*v*_ vesicles would undergo exocytosis directly before having the chance to transition to more advanced stages. Models where low *p*_*v*_ vesicles never undergo exocytosis directly would predict that the complete sequence of priming transitions is even faster; specifically, faster than ${\hat{\beta }}_{SS100}=13.5/s$ (but this seems unlikely given the results in Fig 5).

#### Lemma 6 Similar timing of recruitment to low and high *p*_*v*_ release sites

Reports of experiments on calyces of Held at earlier developmental stages have suggested that recruitment of vesicles with low *p*_*v*_ is much faster than vesicles with high *p*_*v*_[28]. We saw no evidence for this.

That is, the increase in release elicited by the frequency jumps is isolated in Fig 3D. The increase in the steady state response was expected as a direct consequence of more vacancies in the RRP because full sites would occlude recruitment of new vesicles; *e.g.*, ${\hat{\alpha }}_{t}\cdot \left(RRP_{0}-RRP_{t}\right)$ in Eq 1 would be greater because *RRP*_*t*_ would be lesser. Thus, an estimate for the recruitment rate for a pure population of vesicles with low *p*_*v*_ could be calculated by dividing the increase in the steady state by the quantity elicited by release at 300*Hz* that was not released at 100*Hz* (*i.e.*, *RRP*_*SS*100_).

The precise value of the estimate would depend on the specific assumptions used to deconvolve the increase in bulk vesicle recruitment from the total increase; the possibilities are explained in the Results for Fig 4. The green lines in Figs 3G and 4B assume bulk recruitment was maximal from the start; in this case, vesicles with low *p*_*v*_ would be recruited at a unitary rate of 5.8/*s* ± 0.6/*s*(*n* = 20), which is an upper bound. Conversely, the brown line assumes that bulk recruitment did not increase until after the RRP was exhausted, and produced a lower bound of 3.3/*s* ± 0.2/*s*.

Notably, the range was likely overestimated because some of the increase in the steady state release could have been caused by acceleration of recruitment to release sites that were already empty during the preceding 100*Hz* stimulation. Despite being an overestimate, however, the range is similar to the range of 3.5/*s* ± 0.15/*s* to 5.4/*s* ± 0.4/*s* for the mixed population of vesicles with low and high high *p*_*v*_ that was calculated with an identical analysis from the responses when 300*Hz* stimulation was initiated from rest. This analysis confirms that the vesicles remaining within the RRP during 100*Hz* stimulation (*i.e.*, the ones with low *p*_*v*_), and the vesicles with high *p*_*v*_, were recruited to the RRP with similar timing during ongoing 300*Hz* stimulation. The logic does not depend on any particular model. The analysis rules out a 10-fold or even 2-fold difference in the timing, but does not have the resolution to rule out the small difference of ∼100*ms* predicted for sequential models in **Lemma 5**.

#### Lemma 7 Parallel models: On target prediction of numbers of low vs high *p*_*v*_ release sites

If vesicles with low and high *p*_*v*_ are recruited to separate types of release sites, the RRP would be subdivided into two stable subdivisions and versions of Eq 1 would apply to each separately. Specifically for the subdivision containing vesicles with low *p*_*v*_:

$$\begin{array}{c} \frac{dLpD_{t}}{dt}={\hat{\alpha }}_{LpD,t}\cdot \left(LpD_{0}-LpD_{t}\right)-{\hat{\beta }}_{LpD,t}\cdot LpD_{t} \end{array}$$ (7)

where: *LpD*_*t*_ is the quantity of transmitter contained in the low *p*_*v*_ subdivision (*i.e.*, Low ${\underline{p}}_{v}$ Division) at time *t*; ${\hat{\alpha }}_{LpD,t}$ is the average unitary rate of vesicle recruitment to all release sites in *LpD*_*t*_ that are empty; and ${\hat{\beta }}_{LpD,t}$ is the average unitary rate of transmitter release from all *LpD*_*t*_ sites that are full.

When the number of vesicles with low *p*_*v*_ is in a steady state, $\frac{dLpD_{t}}{dt}=0$, and therefore:

$$\begin{array}{c} LpD_{SS100}=\frac{{\hat{\alpha }}_{LpD,SS100}}{{\hat{\alpha }}_{LpD,ss100}+{\hat{\beta }}_{LpD,ss100}}\cdot LpD_{0} \end{array}$$ (8)

where *t* = *SS*100 when the low *p*_*v*_ subdivision is in a steady state during 100*Hz* stimulation.

The *p*_*v*_ = 4.7% value from **Lemma 3** is converted to ${\hat{\beta }}_{LpD,SS100}=4.7/s$ by multiplying by the stimulating frequency. And ${\hat{\alpha }}_{LpD,SS100}$ was likely only marginally different than ${\hat{\alpha }}_{t}=3.6/s$ from Fig 7D because ${\hat{\alpha }}_{t}$ only increased a small amount when the stimulating frequency was increased from 100 to 300*Hz* (see second order considerations, below, for the full range of possibilities). Therefore:

$$\begin{array}{c} LpD_{SS100}=0.43\cdot LpD_{0} \end{array}$$ (9)

which was calculated directly from Eq 8.

From Fig 7B (left panel, checkerboard magenta bar):

$$\begin{array}{c} LpD_{SS100}=0.21\cdot RRP_{0} \end{array}$$ (10)

Combining Eqs 9 and 10 yields:

$$\begin{array}{c} \frac{LpD_{0}}{RRP_{0}}=\frac{0.21}{0.43}=49\;\% \end{array}$$

Thus, so called parallel models predict that about half of release sites would release vesicles with low *p*_*v*_. The conclusion is remarkable because it is extrapolated from estimates of the steady state fullness of the RRP when only ∼20% full, and yet correctly predicts results in previous studies showing that approximately half of readily releasable vesicles are slow-releasing when the RRP is full [17, 46]; *i.e.*, vesicles with low *p*_*v*_ would be slow-releasing because they are released in bulk more slowly over time than vesicles with high *p*_*v*_.

#### Second order corrections

The higher steady state release rate during 300*Hz* compared to 100*Hz* stimulation indicates that the bulk rate of vesicle recruitment to the RRP was faster because the release rate is equivalent to recruitment when the RRP is maintained at a steady state level of fullness; the evidence for a steady state level is in Fig 3G. Three separate mechanisms could have contributed. (1) Constant capacity models predict that at least a part of the increase was simply caused by more vacancies in the RRP because full sites would occlude recruitment of new vesicles; ${\hat{\alpha }}_{t}\cdot \left(RRP_{0}-RRP_{t}\right)$ in Eq 1 would be greater because *RRP*_*t*_ would be lesser. (2) 300*Hz* stimulation might accelerate the recruitment mechanism beyond the level achieved during 100*Hz* stimulation. (3) Vesicle recruitment to low *p*_*v*_ release sites might be intrinsically faster than to high *p*_*v*_ release sites, so freeing up low *p*_*v*_ sites with 300*Hz* stimulation could increase the mean (**Lemma 6** establishes boundary conditions but does not rule out a modest contribution).

The estimate for $\frac{LpD_{0}}{RRP_{0}}$ above assumed that ${\hat{\alpha }}_{LpD,SS100}={\hat{\alpha }}_{SS100}$, which would be accurate if the increase was caused by any combination of the first two mechanisms. However, ${\hat{\alpha }}_{LpD,SS100}>{\hat{\alpha }}_{SS100}$ if the third mechanism played a role, meaning that *LpD*_*SS*100_ > 0.43 ⋅ *LpD*_0_, making $\frac{LpD_{0}}{RRP_{0}}<49\%$.

*H*ow much less? Further analysis showed that the upper bound for ${\hat{\alpha }}_{LpD,SS100}$ would be 5.0/*s*, which assumes that the small increase in ${\hat{\alpha }}_{t}$ in Fig 7D after the frequency jump was wholly caused by intrinsically faster recruitment of vesicles to low *p*_*v*_ sites, and not at all to activity-dependent acceleration of the recruitment mechanism. For this case, vesicle recruitment to newly vacated sites was calculated with the procedure used to generate the blue line in Fig 3G, except using a larger data set, and resulted in *LpD*_*SS*100_ = 0.52 ⋅ *LpD*_0_ (from Eq 8). Combining this with Eq 10:

$$\begin{array}{c} \frac{LpD_{0}}{RRP_{0}}>\frac{0.21}{0.52}=40\;\% \end{array}$$

which matches measurements for the same developmental stage [46].

#### Lemma 8 A posteriori models: Release sites with low *p*_*v*_ are not generated by global inactivation/fatigue

Previous studies suggested the existence of vesicles with low *p*_*v*_ because the time course of RRP depletion during strong stimulation does not follow a single exponential. Instead, the time course is typically fit by the sum of two exponentials, which is consistent with two classes of vesicles with distinct values for *p*_*v*_.

An alternative explanation that was never ruled out would be that the release machinery fatigues after catalyzing the release of half of the vesicles; these are termed *a posteriori* models [31]. Fatigue could occur because of: Ca^2+^-channel inactivation; glutamate activation of presynaptic metabotropic receptors; or some other fatigue of the release machinery caused by processing exocytosis [36, 69]. In any case, fatigue in the release machinery as an explanation of multiple phases of release would be a special case of parallel models where the *p*_*v*_ values would decrease over time during stimulation.

For *a posteriori* models, Eq 9 in **Lemma 7** would be re-interpreted to mean that 43% of the fatigued sites are occupied during steady state 100*Hz* stimulation. But, the experimental results and logic leading to Eq 10 that only ∼21% of all release sites are occupied at 100*Hz* steady state would continue to be valid, which—when taken together with Eq 9—would indicate that approximately half of all release sites maintain a high *p*_*v*_ identity.

This reasoning argues against the possibility that *p*_*v*_ for individual release sites decreases during stimulation, but is similar across all release sites at any given point in time; *i.e.*, as would be expected if an *a posteriori* mechanism were the cause of the multiple phases of release that are observed. The second order corrections in **Lemma 7** only strengthen the reasoning against *a posteriori* models because the corrections would increase the fraction of sites that have intrinsically high *p*_*v*_.

*A posteriori* models can additionally be ruled out simply from the mismatch between the estimate of ${\hat{\beta }}_{SS100}=13.5/s$ in **Lemma 4**, which implies a mean *p*_*v*_ of 13.5% for all vesicles within the RRP, and the mean value for *p*_*v*_ specifically for the vesicles in the standing supply of only 4.7% calculated in **Lemma 3**.

Both lines of reasoning argue that vesicles with a wide range of values for *p*_*v*_ coexist simultaneously within the RRP during steady state 100*Hz* stimulation. Notably, the reasoning does not rule out more complicated models where: (1) only half of release sites are susceptible to fatigue; or (2) release sites flicker back and forth between states with high and low *p*_*v*_ such that approximately half are in each category at any given moment during steady state 100*Hz* stimulation [47, 70].

#### Lemma 9 Estimates of *p*_*v*_ for release sites with intrinsically high release probability

For parallel models, the values for $\frac{LpD_{0}}{RRP_{0}}$ plotted along the x-axis in Fig 9 could be combined with estimates of *p*_*v*, *low*_ and the mean release probability of all of the vesicles in the RRP at the start of stimulation (*i.e.*, *p*_*v*, *total*_) to estimate the mean release probability of the vesicles with intrinsically high release probability (*i.e.*, *p*_*v*, *hi*_):

$$\begin{array}{c} p_{v,hi}=\frac{p_{v,total}-\frac{p_{v,low}\cdot LpD_{0}}{RRP_{0}}}{1-\frac{LpD_{0}}{RRP_{0}}} \end{array}$$

*p*_*v*, *total*_ was extracted from the responses during 300*Hz* stimulation using Eqns 1 and 2; the population mean was 11.8% as documented in the **Results** section. The values for *p*_*v*, *low*_ calculated for times when stimulation was 100*Hz* in **Lemma 3** were likely greater than the value for *p*_*v*, *low*_ at the beginning of stimulation before the induction of facilitation, and could be used to calculate a lower boundary for *p*_*v*, *hi*_. The complimentary upper boundary could be calculated by assuming that *p*_*v*, *low*_ = 0 at the beginning of stimulation.

With these parameters, *p*_*v*, *hi*_ would be between 17.3 ± 2.1% and 24.5 ± 3.5%. The true value would likely be somewhere in the interior of the range. The upper bound seems unlikely because the assumption that *p*_*v*, *low*_ = 0 seems to be inconsistent with the correlation between the standing steady state level of reluctant vesicles during 100*Hz* stimulation and paired pulse facilitation plotted in Fig 9. At the other end of the range, the assumption that *p*_*v*, *low*_ is not enhanced by 100*Hz* stimulation does not seem to be compatible with the robust facilitation seen after switching to 300*Hz* (*e.g.*, Fig 3G).

The calculation pertains specifically to parallel models. However, the result would be similar for sequential models because: (1) $\frac{LpD_{0}}{RRP_{0}}$, which is the relative size of the SRP at the beginning of stimulation, has been measured directly and is similar to the estimates extracted here assuming a parallel arrangement (see **Lemma 7**); and (2) the estimate of *p*_*v*, *low*_ in **Lemma 3** pertains to all models covered by Eq 1.

## Supporting Information

S1 Fig RRP depletion in 1.2 *mM* Ca^2+^. **A-D.** Experiments were similar to Fig 2 except extracellular Ca^2+^ was switched from 2 *mM* to 1.2 *mM* and then back to 2 *mM*. Stimulation was 300 *Hz* for 200 *ms* in 2 *mM* Ca^2+^ (blue circles in **Panel(A)**), then 300 *Hz* for 200 *ms* **Panel(B)** and 100 *Hz* for 500 *ms* **Panel(C)** in 1.2 *mM* Ca^2+^, and then again 300 *Hz* for 200 *ms* after returning to 2 *mM* Ca^2+^ (green squares in **Panel(A)**). Trials were conducted in sets of 3 separated by 1 *min* rest intervals and were averaged together at the level of raw data before further analysis. Segments were integrated and then normalized by the mean of the integral of the first 3.33 *ms* segments during stimulation in 2 *mM* Ca^2+^. Data were only accepted for further analysis if recovery of the integrated response during 300 *Hz* stimulation was to within 6%; mean recovery was 100% ± 2%(*n* = 4). Magenta lines in **Panels (A)—(C)** demarcate the fraction of responses attributed to release from vesicles recruited to the RRP after the onset of stimulation using Eqs 1 and 2; the only free parameter was *τ* = 10 action potentials in Eq 2, which was extracted from experiments summarized in Fig 7B. The premises are that the RRP has a fixed capacity and that the train stimulation drove RRP fullness to a steady state level in each case. The steady state values are larger in **Panel (C)** compared to **Panels (A)—(B)** because the segments were 3-fold longer; the steady state rate of transmitter release was lower during 100 *Hz* *vs* 300 *Hz* stimulation as expected. **D.** Cumulative plots *vs* time for responses in **Panels (A)—(C)**; symbol color and shape are matched, but error bars are omitted. **E.** Estimated fraction of the RRP released by the various stimulation protocols. The results for 2 *mM* and 4 *mM* Ca^2+^ are taken from Fig 7B (solid magenta bars). The results for 1.2 *mM* Ca^2+^ are simply the sum of points in **Panels (B) and (C)** divided by the sum in **Panel (A)** after first subtracting the magenta lines.
(PDF)
