# Correction: A Well-Defined Readily Releasable Pool with Fixed Capacity for Storing Vesicles at Calyx of Held

**DOI:** 10.1371/journal.pcbi.1013339

**Published:** 2025-07-31

**Authors:** Kashif Mahfooz, Mahendra Singh, Robert Renden, John F. Wesseling

The hyperlink in the Data Availability statement is incorrect. The correct hyperlink in the Data Availability statement is: https://doi.org/10.5061/dryad.rh97t.
