# Supplementary material for: A Well-Defined Readily Releasable Pool with Fixed Capacity for Storing Vesicles at Calyx of Held
Source: PLoS Comput Biol. 2016 Apr 1;12(4):e1004855. doi: 10.1371/journal.pcbi.1004855 (PMC4818018; doi:10.1371/journal.pcbi.1004855)
Supplement: S1 Fig — A-D. Experiments were similar to Fig 2 except extracellular Ca2+ was switched from 2 mM to 1.2 mM and then back to 2 mM. Stimulation was 300 Hz for 200 ms in 2 mM Ca2+ (blue circles in Panel(A)), then 300 Hz for 200 ms Panel(B) and 100 Hz for 500 ms Panel(C) in 1.2 mM Ca2+, and then again 300 Hz for 200 ms after returning to 2 mM Ca2+ (green squares in Panel(A)). Trials were conducted in sets of 3 separated by 1 min rest intervals and were averaged together at the level of raw data before further analysis. Segments were integrated and then normalized by the mean of the integral of the first 3.33 ms segments during stimulation in 2 mM Ca2+. Data were only accepted for further analysis if recovery of the integrated response during 300 Hz stimulation was to within 6%; mean recovery was 100% ± 2%(n = 4). Magenta lines in Panels (A)—(C) demarcate the fraction of responses attributed to release from vesicles recruited to the RRP after the onset of stimulation using Eqs 1 and 2; the only free parameter was τ = 10 action potentials in Eq 2, which was extracted from experiments summarized in Fig 7B. The premises are that the RRP has a fixed capacity and that the train stimulation drove RRP fullness to a steady state level in each case. The steady state values are larger in Panel (C) compared to Panels (A)—(B) because the segments were 3-fold longer; the steady state rate of transmitter release was lower during 100 Hz vs 300 Hz stimulation as expected. D. Cumulative plots vs time for responses in Panels (A)—(C); symbol color and shape are matched, but error bars are omitted. E. Estimated fraction of the RRP released by the various stimulation protocols. The results for 2 mM and 4 mM Ca2+ are taken from Fig 7B (solid magenta bars). The results for 1.2 mM Ca2+ are simply the sum of points in Panels (B) and (C) divided by the sum in Panel (A) after first subtracting the magenta lines. (PDF) [file pcbi.1004855.s001.pdf]

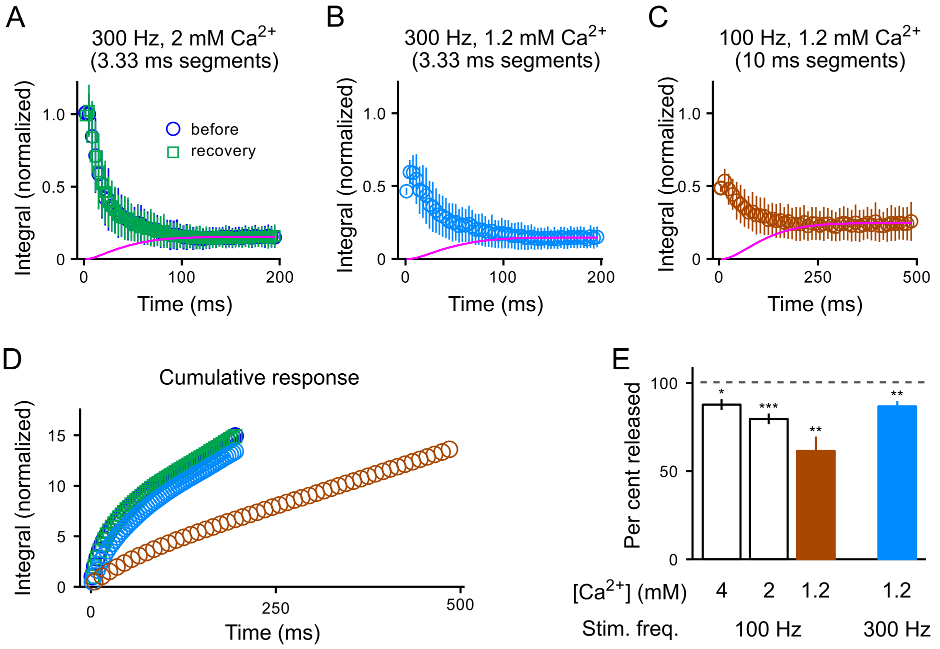

S1 Supporting Figure. RRP depletion in 1.2 mM Ca^{2+}. A-D. Experiments were similar to **Fig. 2** except extracellular Ca^{2+} was switched from 2 mM to 1.2 mM and then back to 2 mM. Stimulation was 300 Hz for 200 ms in 2 mM Ca^{2+} (blue circles in **Panel (A)**), then 300 Hz for 200 ms **Panel (B)** and 100 Hz for 500 ms **Panel (C)** in 1.2 mM Ca^{2+}, and then again 300 Hz for 200 ms after returning to 2 mM Ca^{2+} (green squares in **Panel (A)**). Trials were conducted in sets of 3 separated by 1 min rest intervals and were averaged together at the level of raw data before further analysis. Segments were integrated and then normalized by the mean of the integral of the first 3.33 ms segments during stimulation in 2 mM Ca^{2+}. Data were only accepted for further analysis if recovery of the integrated response during 300 Hz stimulation was to within 6 %; mean recovery was 100 % ± 2 % ($n = 4$). Magenta lines in **Panels (A) - (C)** demarcate the fraction of responses attributed to release from vesicles recruited to the RRP after the onset of stimulation using Eqns 1 and 2; the only free parameter was τ = 10 action potentials in Eqn 2, which was extracted from experiments summarized in **Fig. 7B**. The premises are that the RRP has a fixed capacity and that the train stimulation drove RRP fullness to a steady state level in each case. The steady state values are larger in **Panel (C)** compared to **Panels (A) - (B)** because the segments were 3-fold longer; the steady state rate of transmitter release was lower during 100 Hz *vs* 300 Hz stimulation as expected. **D.** Cumulative plots *vs* time for responses in **Panels (A) - (C)**; symbol color and shape are matched, but error bars are omitted. **E.** Estimated fraction of the RRP released by the various stimulation protocols. The results for 2 mM and 4 mM Ca^{2+} are taken from **Fig. 7B** (solid magenta bars). The results for 1.2 mM Ca^{2+} are simply the sum of points in **Panels (B) and (C)** divided by the sum in **Panel (A)** after first subtracting the magenta lines.

Equations (same as in article)

$$\frac{dRRP_t}{dt} = \hat{\alpha}_t \cdot (RRP_0 - RRP_t) - \hat{\beta}_t \cdot RRP_t \qquad (1)$$

$$\hat{\alpha}_t = \hat{\alpha}_{max} \cdot \left(1 - e^{\frac{-t}{\tau}}\right) \qquad (2)$$
